# Presence of potentially novel *Helicobacter pylori*-like organisms in gastric samples from cats and dogs

**DOI:** 10.1186/s13567-023-01223-4

**Published:** 2023-10-17

**Authors:** Emily Taillieu, Sofie De Bruyckere, Christophe Van Steenkiste, Koen Chiers, Freddy Haesebrouck

**Affiliations:** 1 Department of Pathobiology, Pharmacology and Zoological Medicine, Faculty of Veterinary Medicine, Ghent University, Merelbeke, Belgium; 2 Department of Gastroenterology and Hepatology, University Hospital Antwerp, Antwerp University, Edegem, Belgium; 3 Department of Gastroenterology and Hepatology, General Hospital Maria Middelares, Ghent, Belgium

**Keywords:** Gastric *Helicobacter* species, non-*Helicobacter pylori Helicobacter* species, dog, cat

## Abstract

While seven gastric non-*Helicobacter pylori Helicobacter* (NHPH) species are known to commonly colonize the stomach of cats and dogs, the potential of *H. pylori* and *H. pylori*-like organisms to infect animals remains controversial and was investigated in this study using gastric samples of 20 cats and 27 dogs. A *Helicobacter* genus-specific *16 S rRNA* PCR assay, *H. pylori*-specific *ureAB* and *glmM* PCR assays and a nested PCR detecting *23 S rRNA* in a *Helicobacter* genus-specific manner in a first round of PCR and a *H. pylori*-specific manner in a second round, were performed in combination with sequencing. Histopathological and anti-*Helicobacter* immunohistochemical evaluations were also performed. Based on *16 S rRNA* sequence analysis, 39/47 animals (83%) appeared infected with canine/feline gastric NHPHs in the corpus and/or antrum. *H. pylori*-specific *ureAB* amplicons were obtained in samples of 22 stomachs (47%). One canine antrum sample positive in the *ureAB* assay was also positive in the *H. pylori*-specific *glmM* assay. While 36/47 (77%) animals had a positive sample in the first round of the nested *23 S rRNA* PCR assay, all samples were negative in the second round. Sequence analysis of obtained amplicons and immunohistochemistry point towards the presence of unidentified *H. pylori*-like organisms in cats and dogs. Histopathological examination suggests a low pathogenic significance of the gastric *Helicobacter* spp. present in these animals. In conclusion, cats and dogs may be (co-)infected with gastric *Helicobacter* organisms other than the known gastric NHPHs. Culture and isolation should be performed to confirm this hypothesis.

**Supplementary Information:**

The online version contains supplementary material available at 10.1186/s13567-023-01223-4.

## Introduction

Within the genus *Helicobacter*, *Helicobacter pylori* (*H. pylori*) is by far the most widely known species. It is a gastric *Helicobacter* species residing in the stomach of humans since the existence of the modern human [1]. At present, it is known to infect more than 50% of the human population worldwide, with a higher prevalence in low-income countries compared to developed countries [2]. Marshall and Warren were the first to isolate this bacterium from a human gastric biopsy, dating from 1982, and to provide the evidence for an association between *H. pylori* infection and gastric disease [3]. *H. pylori* infections can result in asymptomatic carriership, but may also cause acute and chronic gastritis, peptic ulcer disease, and less often gastric carcinoma and mucosa-associated lymphoid tissue (MALT) lymphoma [4].

Besides *H. pylori*, there are 52 other validly published *Helicobacter* species, called non-*Helicobacter pylori Helicobacter* (NHPH) species, of which 16 that also prefer to colonize the gastric mucosa of their specific host [5]. *H. ailurogastricus*, *H. baculiformis*, *H. bizzozeronii*, *H. cynogastricus*, *H. felis*, *H. heilmannii* s.s. and *H. salomonis* belong to a group of canine and feline associated gastric NHPHs. In several case reports and cohort studies, these have been described to colonize the stomach of domestic and stray dogs and cats. Some of these reports link NHPH infections to a higher susceptibility for feline gastric MALT lymphoma [6] or more severe gastritis in dogs [7], however, most of them point towards a low pathogenic impact of gastric NHPHs in cats and dogs [8–17]. This low pathogenic impact may be explained by the fact that these canine/feline associated gastric NHPHs have coevolved with their host far before domestication of either cats or dogs [18]. Most of these dog- and cat-associated NHPHs may also infect humans, which may result in gastric disease [19, 20].

Interestingly, there have been sporadic reports of natural *H. pylori* infections in cats and dogs. Already in 1994, Handt et al. [21], reported the isolation of *H. pylori* from the gastric tissue of 6 cats, confirmed by morphologic and biochemical evaluations, fatty acid analysis and *16 S rRNA* sequence analysis and suggested the detection of *H. pylori* based on histopathological evaluation in an additional 15 cats. They hypothesized a causal role of *H. pylori* for the development of lymphofollicular gastritis in domestic cats and a zoonotic component for the transmission of *H. pylori* in humans. Later, these conclusions were challenged by El-Zaatari et al. [22], among others, who reported that owning cats is not associated with a higher risk of acquiring *H. pylori* infections, but sporadic *H. pylori* infections in cats are likely cases of anthroponoses. More recently, Kubota-Aizawa et al. [23] reported the detection of infections with identical *H. pylori* strains in a woman and her two dogs, based on sequence analysis of partial *ureAB* sequences. Here too, the mode of transmission was considered to be anthroponotic.

Of note, Krakowka et al. [24] have reported the presence of *H. pylori*-like organisms in pigs, which are structurally and immunologically closely related to, however antigenically distinct from, *H. pylori*, and morphologically distinct from *H. suis*, which is a well-known pathogen in pigs. Such pig associated *H. pylori*-like organisms have also been described by Cortez Nunes et al. [25] who detected *Helicobacter* species in DNA samples of gastric tissue of 36 out of 71 pigs (50.7%) for which amplification in a *ureAB* gene based *H. pylori*-specific PCR assay was achieved (as confirmed by sequencing), but not in a *glmM* gene based *H. pylori*-specific PCR assay.

Consequently, the aim of the current study was to further investigate the presence of *H. pylori*(-like organisms) in cats and dogs.

## Materials and methods

### Sample collection

Samples were collected in the autopsy room of the Laboratory of Veterinary Pathology of the Faculty of Veterinary Medicine in Merelbeke, Belgium, during a period dating from November 9th, 2022 until December 9th, 2022. In total, 47 animals, all from different owners, were included, of which 20 cats and 27 dogs. The stomach of each animal was opened along the greater curvature and the insides were rinsed with tap water. From each stomach, 3 samples were taken from both the antrum and the corpus using 8 mm disposable biopsy punches and/or autoclaved scissors and tweezers for DNA extraction. Also using autoclaved scissors and tweezers, additional biopsy samples from the antrum and corpus were taken for histology purposes. In 33 cases where stool was present in the colon, a stool sample was also collected. The samples were stored at −20 °C until further processing.

DNA of 24 more dog stool samples from another study (unpublished results) were also included. These were fresh stool samples collected from alive dogs, either healthy or suffering from idiopathic epilepsy. These dogs were all from different owners.

### DNA extraction, PCR assays and sequencing

#### DNA extraction

DNA was extracted from the gastric biopsy samples of the antrum and the corpus, separately, using the DNeasy Blood & Tissue kit (Qiagen, Hilden, Germany) according to the manufacturer’s instructions. As for the stool samples, DNA was extracted using the QIAamp PowerFecal Pro DNA Kit (Qiagen, Hilden, Germany) according to the manufacturer’s instructions.

**Table 1:** **Details on PCR primers and protocols**

PCR assay	Target gene	Primer	Primer sequence	Thermocycling conditions	Amplicon size (bp)	Refs.
*Helicobacter* genus	*16 S rRNA*	Hcom1	FW (5′-GTAAAGGCTCACCAAGGCTAT-3′)	5′ 94 °C 40 × (1′ 94 °C + 1′ 63 °C + 1′72 °C) 5′ 72 °C	390	[26]
		Hcom2	RV (5′-CCACCTACCTCTCCCACACTC-3′)			
*H. pylori*	*UreAB*	BFHpyl_F1	FW (5′-AAAGAGCGTGGTTTTCATGGCG-3′)	4′ 95 °C 45 × (30″ 94 °C + 30″ 59 °C + 1′ 72 °C) 10′ 72 °C	217	[27]
		BFHpyl_R1	RV (5′-GGGTTTTACCGCCACCGAATTTAA-3′)			
*H. pylori*	*glmM* (*UreC*)	Hpy3F	FW (5′-TTATCGGTAAAGACACCAGAAA-3′)	15′ 94 °C 45 × (45″ 94 °C + 45″ 58 °C + 45″ 72 °C) 7′ 72 °C	144	[23]
		Hpy3R	RV (5′-ATCACAGCGCATGTCTTC-3′)			
First PCR of nested PCR for *H. pylori*	*23 S rRNA*	Hp23S 1835 F	FW (5′-GGTCTCAGCAAAGAGTCCCT-3′)	2′ 95 °C 5 × (30″ 94 °C + 30″ 57 °C + 30″ 72 °C) 30 × (15″ 94 °C + 15″ 57 °C + 20″ 72 °C) 5′ 72 °C	493	[28]
		Hp23S 2327R	RV (5′CCCACCAAGCATTGTCCT-3′)			
Second PCR of nested PCR for *H. pylori*		Hp23S 1942 F	FW (5′-AGGATGCGTCAGTCGCAAGAT-3′)	2′ 95 °C 15 × (10″ 94 °C + 20″ 63 °C) 5′ 72 °C	367	
		Hp23S 2308R	RV (5′-CCTGTGGATAACACAGGCCAGT-3′)			

### PCR assays

#### *Helicobacter* genus-specific *16 S rRNA* PCR assay

A *Helicobacter* genus-specific PCR assay was performed as previously described [20]. Details on the primer sequences and thermocycling conditions can be found in Table 1. As a positive control, genomic DNA of the *H. suis* strain HS5 was used. For visualization and analysis of the PCR assays, 5 µL of each PCR product was analyzed through gel electrophoresis in 1.5% agarose (AGRMP-RO Roche, Merck KGaA, Darmstadt, Germany) with Midori Green (NIPPON Genetics, Düren, Germany) in TBE buffer (VWR Life Science, Amsterdam, The Netherlands). GeneRuler 100 bp Plus DNA Ladder (Thermo Scientific™ SM0323) was used as a weight marker. Images were acquired on a UV transilluminator (UVP PhotoDoc-it Imaging Systems, Fisher Scientific, Hampton, NH, USA).

#### *Helicobacter pylori*-specific PCR assays

Two different *Helicobacter*-specific PCR assays were performed, one based on the *ureAB* gene and another based on the *glmM* gene [20]. Details on the primer sequences and thermocycling conditions can be found in Table 1. As a positive control, genomic DNA of the *H. pylori* strain SS1 was used. For visualization and analysis of the PCR assay, gel electrophoresis was performed as described above.

#### Nested *Helicobacter pylori 23 S rRNA* PCR assay

A nested PCR in two rounds targeting the *23 S rRNA* gene was performed, of which the first PCR is *Helicobacter* genus specific while the second PCR is *H. pylori*-specific. For the first PCR, each PCR reaction volume consisted of 20 µL containing 2.5 mM MgCl_2_ (Promega), 1x GoTaq^®^ Flexi PCR buffer (Promega), 200 µM deoxynucleotide triphosphates (dNTPs) (Bioline), 0.5 µM forward primer, 0.5 µM reverse primer, 0.6 U GoTaq^®^ Flexi DNA polymerase (Promega) and 2 µL of the DNA sample. For the second PCR, each PCR reaction volume consisted of 25 µL containing 2.5 mM MgCl_2_ (Promega), 1x GoTaq^®^ Flexi PCR buffer (Promega), 200 µM deoxynucleotide triphosphates (dNTPs) (Bioline), 0.5 µM forward primer, 0.5 µM reverse primer, 0.75 U GoTaq^®^ Flexi DNA polymerase (Promega) and 1.5 µL of the DNA sample. Details on the primer sequences and thermocycling conditions can be found in Table 1. As a positive control, genomic DNA of the *H. pylori* strain SS1 was used. For visualization and analysis of the PCR assay, gel electrophoresis was performed as described above after each PCR round.

### Sequencing

The PCR products of samples positive in any of the PCR assays were sent to Eurofins Genomics^®^ (Edersberg, Germany) for bidirectional Sanger sequencing, in order to avoid false positive results and confirm the *Helicobacter* species present. Sequencing analysis of amplicons positive for *Helicobacter* genus-specific *16 S rRNA* PCR allows discrimination between *H. suis*, canine and feline associated gastric NHPHs as a group, and *H. pylori*. Sequence editing and assembly of the received amplicon sequences was done using BioNumerics^®^ software (version 7.6.3, Applied Maths, Sint-Martens-Latem, Belgium) and the contig sequences were subjected to the basic local alignment search tool (BLAST) of the National Center for Biotechnology Information (NCBI) using the non-redundant nucleotide database [29]. A cut-off value of 96% was used for average nucleotide identity as a threshold for species delineation [30].

### Alignment and phylogenetic analysis

The evolutionary history was inferred using the Neighbor-Joining method [31]. Multiple sequence alignment was done using ClustalW with a gap opening penalty of 15 and gap extension penalty of 6.66. The bootstrap consensus tree inferred from 1000 replicates was taken to represent the evolutionary history of the taxa analyzed. Branches corresponding to partitions reproduced in less than 50% bootstrap replicates are collapsed. The percentage of replicate trees in which the associated taxa clustered together in the bootstrap test (1000 replicates) are shown next to the branches [32]. The evolutionary distances were computed using the Maximum Composite Likelihood method [33] and are in the units of the number of base substitutions per site. All ambiguous positions were removed for each sequence pair (pairwise deletion option). Evolutionary analyses were conducted in MEGA11 [34].

### Histopathology and immunohistochemistry

#### Histopathology

For histopathology, the biopsies were fixed in formalin and processed for paraffin embedding (formalin-fixed paraffin-embedded (FFPE)). Samples were sectioned at 5 μm and stained with hematoxylin and eosin (H&E) for light microscopic evaluation. Histopathological evaluation was performed by an experienced veterinary histopathologist (KC) in a blinded manner and was based on histopathological standards described by Day et al. [35].

### Immunohistochemistry

For anti-*H. pylori* staining, sections of 5 μm were deparaffinized and hydrated, followed by microwave antigen retrieval in citrate buffer (pH = 6.0; 3.5 min at 850 W, 10 min at 450 W, 30 min cool down). After rinsing with wash buffer, slides were incubated with 3% H_2_O_2_ solution (Agilent Technologies, Santa Clara, California, USA) in methanol (5 min) to block endogenous peroxidase activity. The slides were rinsed once with distilled water and once with wash buffer before they were incubated with a primary antibody (30 min). A polyclonal genus-specific rabbit anti-*H. pylori* antibody (1/250; Agilent Technologies) was used. This commercial antibody was generated from an immunogen prepared from heat-treated cells of the *H. pylori* strain CH-20,426 [36] and is *Helicobacter* genus-specific. The antibody is known to cross-react with *H. suis, H. bizzozeronii* and *H. felis* in gastric tissue from experimentally infected rodents [37–39]. In gastric tissue, it was demonstrated that the sensitivity of the antibody for *H. pylori* was 83.8 ± 11.1% and the specificity 90.0 ± 0.0% [40]. The slides were rinsed with wash buffer and incubated with a peroxidase (HRP) labelled secondary goat anti-rabbit IgG antibody (Agilent Technologies) (30 min). After rinsing twice with wash buffer, the slides were incubated with 3,3’-diaminobenzidine (DAB) solution (Agilent Technologies) (5 min) for color development and rinsed with distilled water. Finally, the slides were counterstained with hematoxylin, dehydrated and mounted. Evaluation of the immunohistochemical stainings was underpinned by an experienced veterinary histopathologist (KC) and performed in a blinded manner.

## Results

### PCR and sequencing results

Absolute frequencies of sequencing-confirmed *Helicobacter* detection in the gastric samples of all cats and dogs included, for each PCR assay, are presented in Table 2. Although enterohepatic *Helicobacter* species were detected in 24 out of the 57 stool samples using the genus *Helicobacter* specific *16 S rRNA* PCR, none of the stool samples were positive for gastric *Helicobacter* species in any of the assays performed.

**Table 2:** **Frequency of *Helicobacter* detection in gastric samples of cats and dogs upon PCR and sequencing**

Animal	Stomach region	Positive for canine/feline associated gastric NHPHs (*16 S rRNA* PCR)^a ^(%)	Positive for *H. pylori*-specific *ureAB* PCR (%)	Positive for *H. pylori*-specific *glmM* PCR (%)	Positive for 1st PCR of nested *Helicobacter 23 S rRNA* PCR^a^(%)	Positive for 2nd PCR of nested *Helicobacter 23 S rRNA* PCR^b^(%)
Cats (n = 20)	Corpus only	3/20 (15)	4/20 (20)	0/20 (0)	5/20 (25)	0/20 (0)
	Antrum only	1/20 (5)	3/20 (15)	0/20 (0)	1/20 (5)	0/20 (0)
	Corpus + antrum	13/20 (65)	2/20 (10)	0/20 (0)	9/20 (45)	0/20 (0)
Dogs (n = 27)	Corpus only	2/27 (7.4)	6/27 (22)	0/27 (0)	2/27 (7.4)	0/27 (0)
	Antrum only	2/27 (7.4)	3/27 (11)	1/27 (3.7)	4/27 (15)	0/27 (0)
	Corpus + antrum	18/27 (67)	4/27 (15)	0/27 (0)	15/27 (59)	0/27 (0)
Total (n = 47)	Corpus and/or antrum	39/47 (83)	22/47 (47)	1/47 (2.1)	36/47 (77)	0/47 (0)

^a^*Helicobacter* genus specific assay.

^b^*H. pylori*-specific assay.

**Figure 1:**
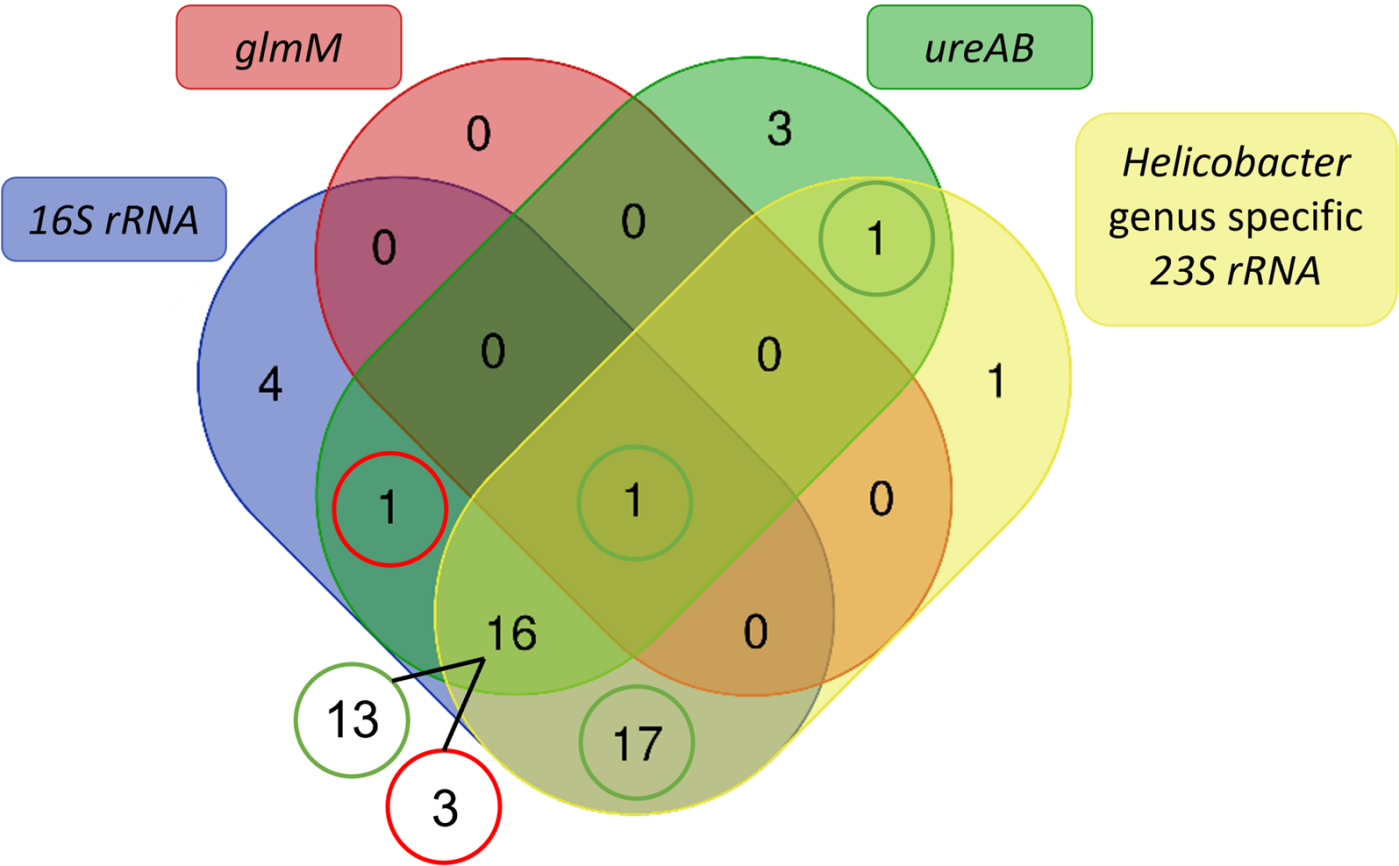
**Venn diagram showing the intersection of PCR and sequencing results at the level of the animals**. Green circles indicate that the assays were positive simultaneously in at least one of both gastric samples of the animal(s); Red circles indicate that the assays were not simultaneously positive in one gastric sample of the animal(s). Created using: [41].

The results of the two-step nested *23 S rRNA* PCR show that 36/47 (77%) animals had a sample positive in the first, *Helicobacter* genus specific PCR, while none of the animals had a sample positive in the second, *H. pylori*-specific PCR. By means of a Venn diagram, the intersection of the PCR results is displayed at the level of the animals (Figure 1). This shows that one dog had a sample, originating from the antrum, that was positive in all PCR assays, except for the *H. pylori*-specific final step of the nested *23 S rRNA* PCR (010DA; see Additional file 1 for detailed results of each sample). However, BLAST results of the amplicon obtained in the *glmM* based *H. pylori*-specific PCR showed a 95.16% identity to *Helicobacter pylori* strain G-Mx-2003-108 chromosome (accession number: CP032044.1) and many other *H. pylori strains* accessible in the NCBI GenBank (including G-Mx-2006-152, FDAARGOS_300, SS1, J99, etc.) and a 96.61% identity and 95% query coverage to *Helicobacter pylori* DNA, complete genome, strain: PMSS1 (accession number: AP017633.1). Taking into account the % identity cut-off value of 96% for species delineation and a desirable query coverage of at least 95%, this sample was considered borderline positive (see Additional file 2 for detailed BLAST results). Except for this sample, no other sample was positive in the *glmM* based PCR, although samples of 22 animals (47%) were positive in the *ureAB* based *H. pylori*-specific assay. In 13/16 animals with a positive result in the *16 S rRNA* PCR, *ureAB* PCR and *Helicobacter* genus specific *23 S rRNA* PCR, these positive results were obtained simultaneously in at least one gastric sample of the individual animal. Regarding the 17 animals which had a positive result in the *16 S rRNA* and *Helicobacter* genus specific *23 S rRNA* PCR, in each case the assays were simultaneously positive in at least one of both gastric samples.

### Phylogenetic analyses

**Figure 2:**
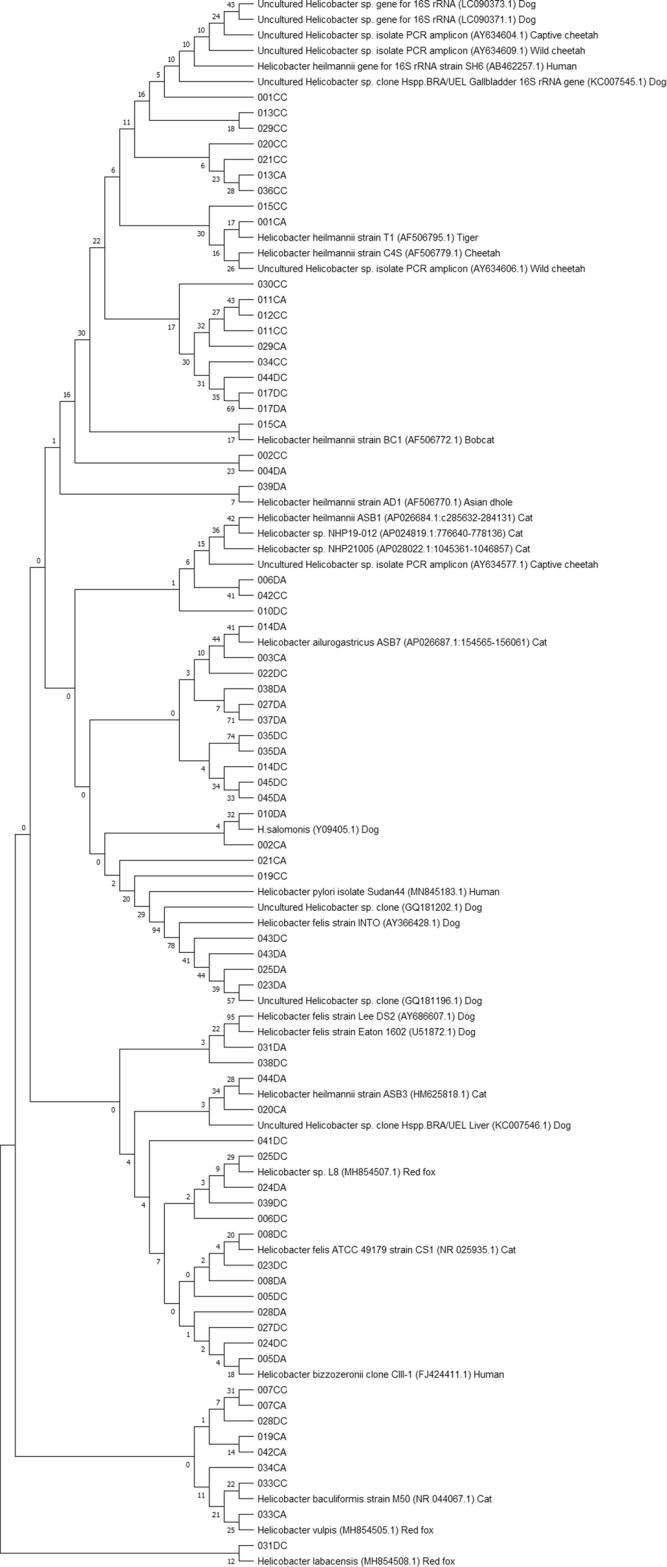
**Neighbor-joining tree based on the comparison of *16 S rRNA *gene sequences**. This phylogenetic tree comprises *16 S rRNA* amplicons obtained in the genus *Helicobacter*-specific *16 S rRNA* PCR and relevant GenBank reference sequences. Sample names include the number assigned to the animal, followed by C or D (= cat or dog) and A or C (= antrum or corpus). Reference sequence names include the *Helicobacter* species, strain, accession number between brackets and host where possible.

**Figure 3:**
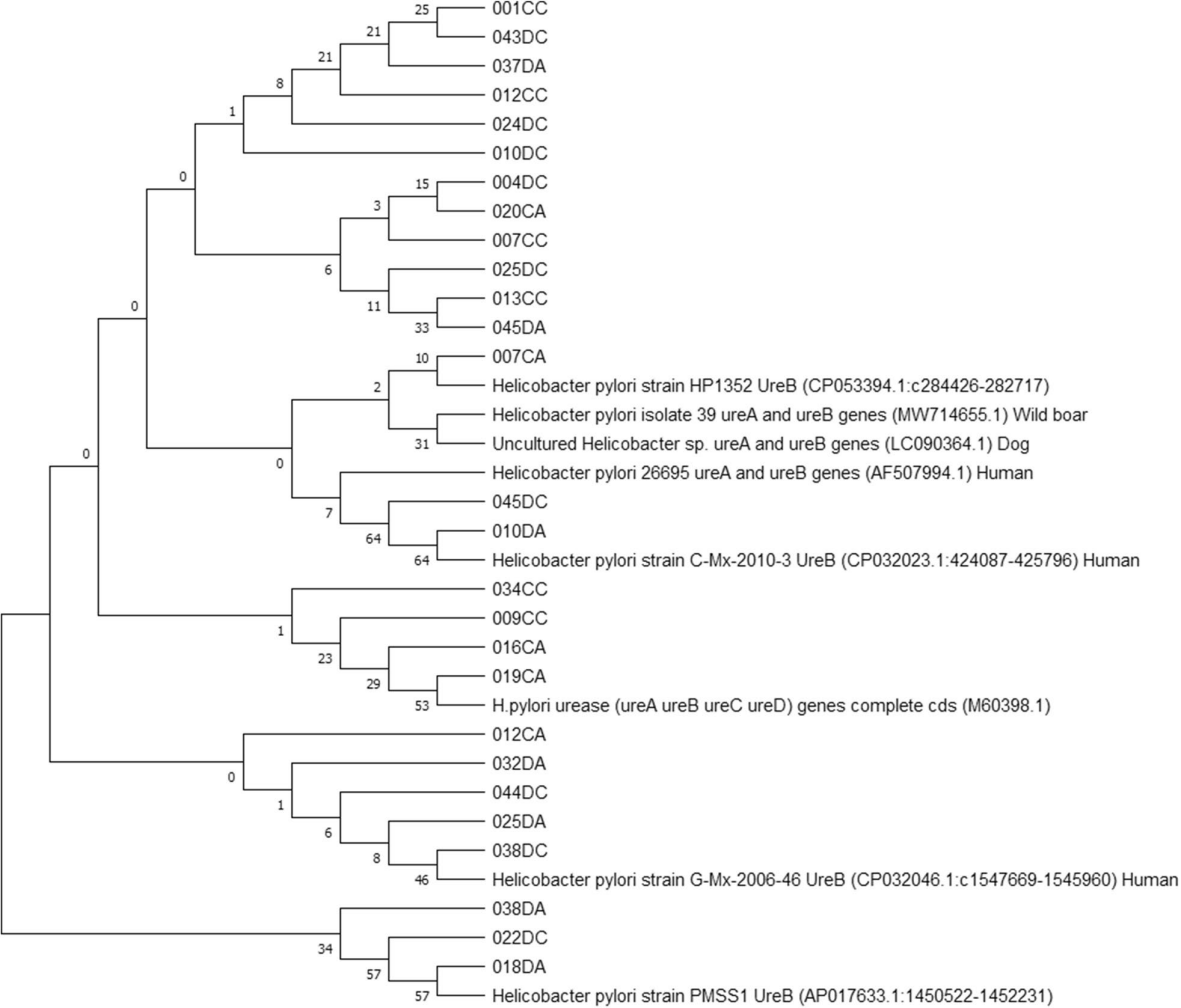
**Neighbor-joining tree based on the comparison of *ureAB *gene sequences**. This phylogenetic tree comprises the *ureAB* amplicons obtained in the *Helicobacter pylori*-specific *ureAB* PCR and relevant GenBank reference sequences. One amplicon sequence was left out of this analysis because sequencing yielded an amplicon that was too short for multiple sequence alignment. Sample names include the number assigned to the animal, followed by C or D (= cat or dog) and A or C (= antrum or corpus). Reference sequence names include the *Helicobacter* species, strain, accession number between brackets and host where possible.

**Figure 4:**
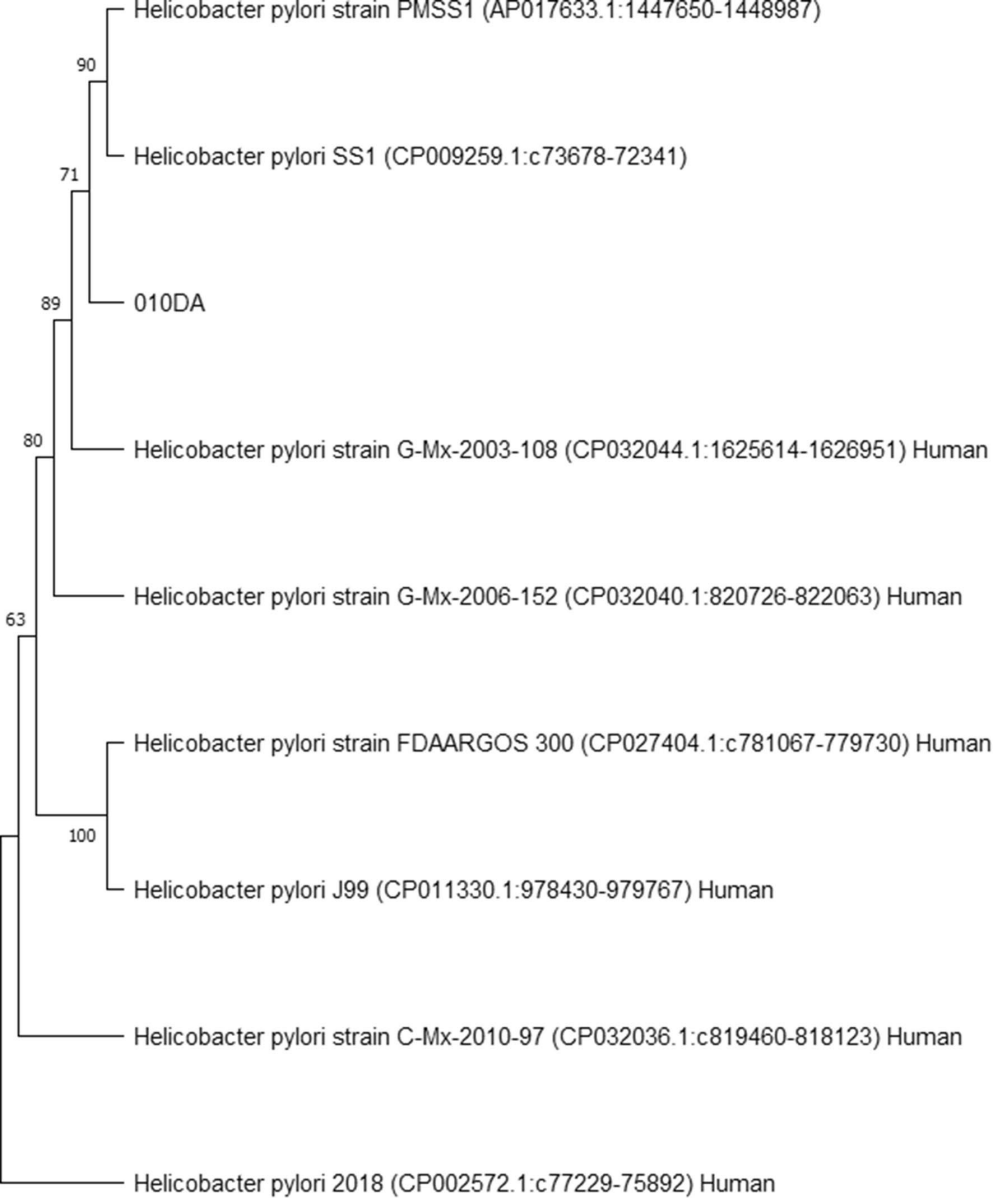
**Neighbor-joining tree based on the comparison of *glmM *gene sequences**. This phylogenetic tree comprises the *glmM* amplicon obtained in the *Helicobacter pylori*-specific *glmM* PCR and relevant GenBank reference sequences. Sample names include the number assigned to the animal, followed by C or D (= cat or dog) and A or C (= antrum or corpus). Reference sequence names include the *Helicobacter* species, strain, accession number between brackets and host where possible.

**Figure 5:**
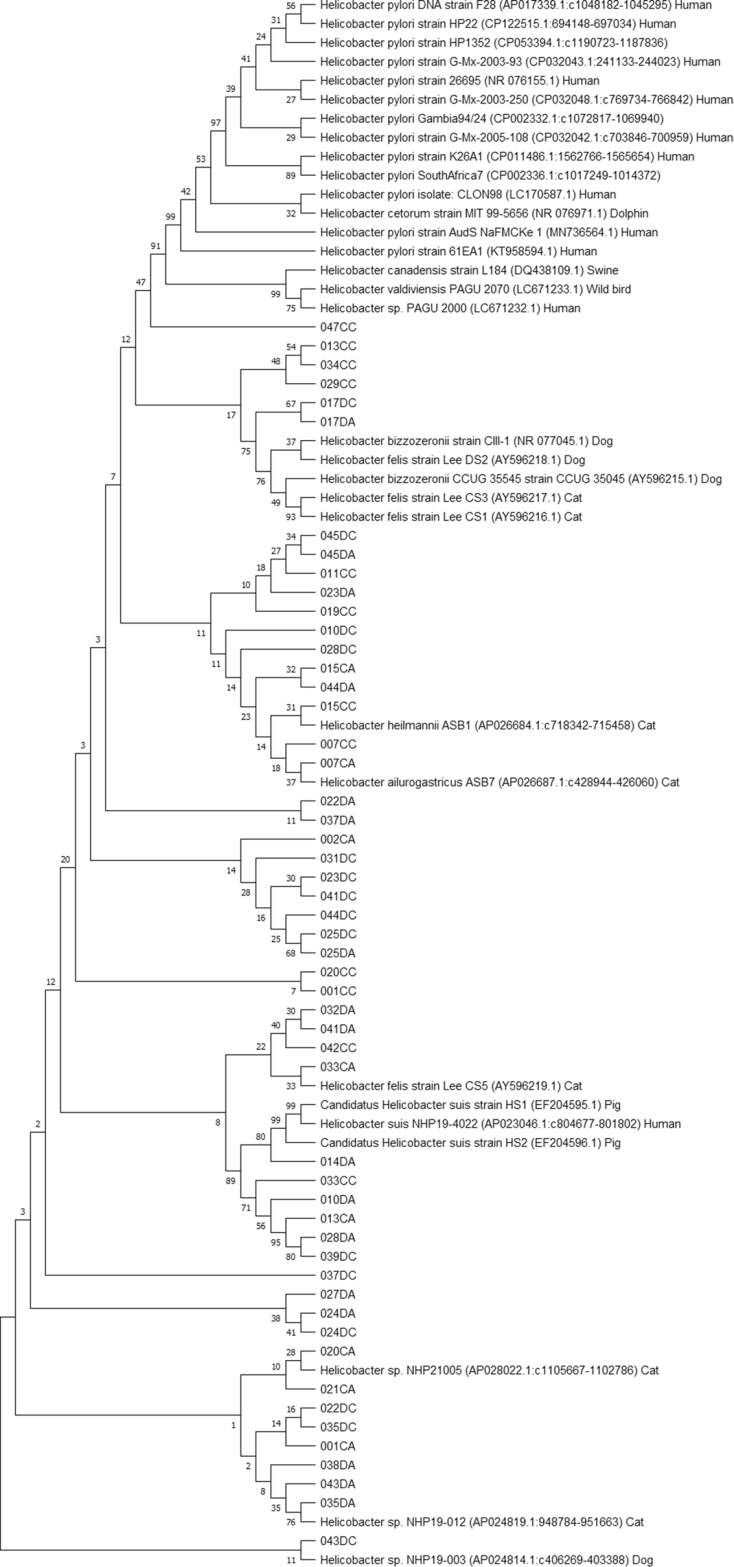
**Neighbor-joining tree based on the comparison of *23 S rRNA *gene sequences**. This phylogenetic tree comprises *23 S rRNA* amplicons obtained in the 1st genus *Helicobacter*-specific PCR of the nested *23 S rRNA* PCR and relevant GenBank reference sequences. Eight amplicon sequences were left out of this analysis because sequencing yielded an amplicon that was too short for multiple sequence alignment. Sample names include the number assigned to the animal, followed by C or D (= cat or dog) and A or C (= antrum or corpus). Reference sequence names include the *Helicobacter* species, strain, accession number between brackets and host where possible.

Phylogenetic analyses were performed using the obtained true positive amplicon sequences in each PCR assay in order to infer evolutionary history of gastric *Helicobacter* species, including potentially novel species, present in the included canine and feline stomach samples (Figures 2, 3, 4 and 5). GenBank reference sequences included in the analyses were obtained from the BLAST results.

### Histopathology and immunohistochemistry

H&E stainings of gastric tissue of 29 animals (61.7%) showed no signs of gastric inflammation in the corpus or antrum (Table 3). These include 23 animals (79.3%) which were positive for the presence of canine/feline associated gastric NHPHs upon PCR (16 in corpus and antrum; four in corpus only; three in antrum only), 14 (48.3%) which had a biopsy specimen positive in the *ureAB* based *H. pylori*-specific PCR (out of which 11 were also positive for gastric NHPHs) (four in corpus and antrum; six in corpus only; four in antrum only) and one (3.4%) which had a biopsy specimen positive in both *H. pylori*-specific PCR assays (cfr. supra).

**Table 3:** **Histopathological evaluation of gastric biopsy specimens based on hematoxylin and eosin staining**

	No gastritis	Mild gastritis	Moderate gastritis	Severe gastritis	Follicular gastritis^a^	Ulcerative
Corpus only	7	0	0	0	6	1 (accompanied by follicular gastritis in antrum)
Antrum only	5	0	1 (accompanied by follicular gastritis and mild mineralization in corpus)	0	8	0
Corpus + antrum	29	1 (probably associated with chronic kidney failure evident by (sub)mucosal mineralization)	0	0	3	0

^a^Follicular gastritis was defined as the presence of at least four basal lymphoid follicles throughout the biopsy section.

In 17 animals (36.2%), follicular gastritis was evident in at least one biopsy specimen. Canine/feline associated gastric NHPHs were detected in 15 (88.2%) of these (14 in corpus and antrum; one in corpus only) and in seven (41.2%), at least one gastric biopsy specimen was positive in the *ureAB* based *H. pylori*-specific PCR (out of which 6 were also positive for gastric NHPHs) (one in corpus and antrum; four in corpus only; two in antrum only).

**Figure 6:**
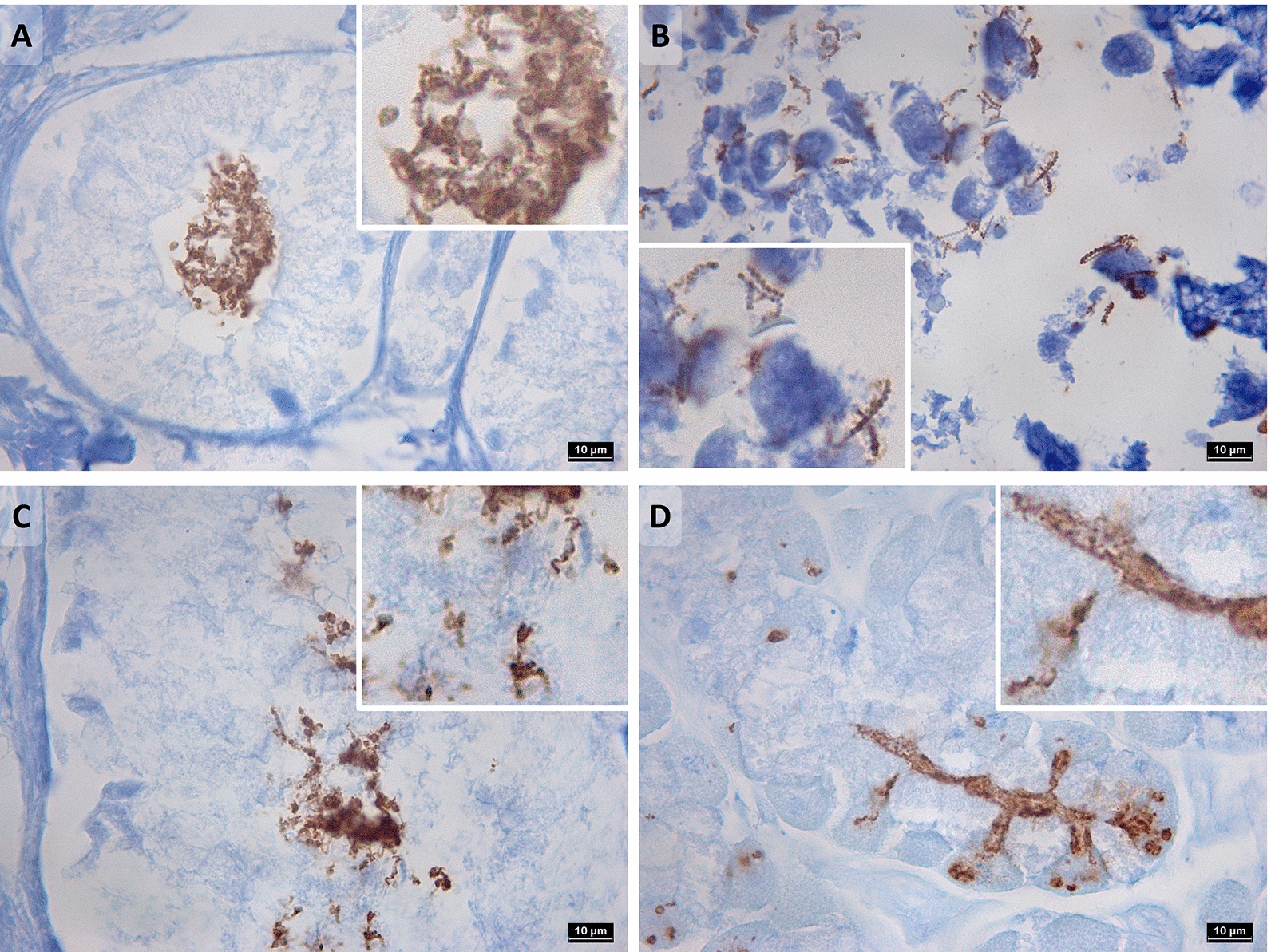
**Anti-*Helicobacter*immunohistochemistry**.** A**,** B** Long spiral-shaped gastric non-*Helicobacter pylori Helicobacter* species, detected in gastric biopsies 044DA (dog, antrum) and 007CC (cat, corpus), respectively. Total magnification ×1000.** C**,** D ***H. pylori*-like organisms detected in gastric biopsies 037DA (dog, antrum) and 043DC (dog, corpus), respectively. Total magnification ×1000.

Anti-*Helicobacter* immunohistochemistry (IHC) was performed in case an animal had a gastric biopsy specimen positive in at least one *H. pylori*-specific PCR assay (i.e. 22 animals positive in the *glmM* and/or *ureAB* based assay). IHC confirmed the presence of the typically long, spiral-shaped gastric NHPHs in 14 of the 18 cases positive in the *16 S rRNA* PCR (Figures 6A, B). Indications for the presence of organisms with a *H. pylori*-like morphology (short, curve- or s-shaped) were found in 14 cases (Figures 6C, D).

## Discussion

The current results support that there may still be novel, uncultured and therefore uncharacterized, gastric *Helicobacter* species (co-)residing in the stomach of cats and dogs. By means of elegant gene admixture analyses performed by Smet et al. [18], it has been shown that intra- and interspecies gene exchange is not uncommon within the group of gastric *Helicobacter* species, especially considering canine/feline associated gastric NHPHs. Since these species are able to co-reside in the canine/feline stomach, this is a logical consequence. *H. cynogastricus* and *H. baculiformis* have even been identified as hybrid species considering the significant amount of DNA originating from *H. felis*. Also, genetic exchange from *H. suis* to *H. heilmannii* s.s. and *H. ailurogastricus* was revealed, although they do not share the same host. The latter may support why *H. suis* also contributed to the phylogenetic analysis results of the *23 S rRNA* PCR amplicon sequences. The current results from *H. pylori*-specific PCR and sequencing analyses and those from partial *ureAB* sequence analyses performed by Kubota-Aizawa et al. [23] in samples of two dogs, may indicate a possibility of anthroponotic *H. pylori* infection and opportunities for genetic exchange between gastric *Helicobacter* species with human and animal hosts. The fact that no samples were positive in the *H. pylori*-specific final step of the nested *23 S rRNA* PCR and only one sample was (borderline) positive in the *H. pylori*-specific *glmM* PCR, indicates that the species detected in these samples are not simply the known human associated *H. pylori*. As no information on the owners and only limited information on the included animals was available for this study, it was not possible to infer any possible transmission route hypotheses.

In the case of dog 010, there are clear indications for the presence of a *Helicobacter* species with a *ureB* and *glmM* sequence showing highest similarity with *H. pylori* sequences obtained from human hosts. Histopathological evaluations showed no signs of inflammation in the corpus or antrum and no macroscopic signs of gastric disease were noted in this dog’s autopsy report. However, anti-*Helicobacter* IHC did show the sporadic presence of possible *H. pylori*-like organisms, although the staining was not as clear compared to certain biopsies taken from other animals in this study due to autolysis.

Overall, this study points towards a low pathogenic significance of the gastric *Helicobacter* spp. detected in the included animals, since the frequency with which canine/feline associated gastric NHPHs and *H. pylori*-like organisms were detected in the PCR assays was similar for animals without signs of gastric inflammation and animals presenting with follicular gastritis upon histopathological evaluation. This supports the current hypothesis that canine/feline associated gastric NHPHs are highly adapted to the colonization niche of their natural hosts under normal conditions [18, 42].

In Figure 6, photographs of the IHC stainings of biopsies 037DA and 043DC were chosen to represent the presence of *H. pylori*-like organisms since these demonstrated the morphology of *H. pylori* most clearly. In retrospect, the *H. pylori*-specific *ureAB* sequences obtained from these biopsies showed highest similarity to a partial coding sequence deposited in the NCBI GenBank database described as “*Helicobacter pylori* isolate 39 urease subunit A and urease subunit B genes” (accession number: MW714655.1), which was obtained from a stomach sample of a wild boar and the organism was later also defined as a *H. pylori*-like organism based on PCR and sequencing analysis [25].

Since both the *16 S rRNA* PCR assay and the first PCR of the nested *23 S rRNA* PCR are genus *Helicobacter*-specific and multiple gastric NHPHs may have co-resided in the animals’ stomachs, the phylogenetic trees constructed with the amplicon sequences obtained from these assays cannot be interpreted unambiguously. However, some samples contained *23 S rRNA* sequences most closely related to *Helicobacter* sp. NHP21005 DNA (AP028022.1), *Helicobacter* sp. NHP19-012 DNA (AP024819.1) and *Helicobacter* sp. NHP19-003 DNA (AP024814.1), of which the latter two have been described as a novel *Helicobacter* sp. isolated from a cat and from a dog, respectively (unpublished results from Rimbara et al., mentioned in the NCBI GenBank submission form of the respective genomes [43, 44]). From the *16 S rRNA* phylogenetic analysis, it can be deduced that there is great genetic variation in the obtained *16 S rRNA* amplicon sequences, since many amplicons showed highest similarity with gastric *Helicobacter* sequences obtained from cheetahs and other wild cats, red foxes and even humans, besides those obtained from domestic cats and dogs.

Ideally, the presence of these potentially novel canine/feline associated gastric *Helicobacter* species should be confirmed through culture and isolation of the species for in depth genomic and biochemical characterization, which was not possible in the current study. To this end, most probably fresh gastric samples will be required and the medium for culture will need to be optimized. Among other things, it will be necessary to test whether a biphasic medium is required, as is the case for *H. suis*, *H. heilmannii* s.s. and *H. ailurogastricus*, and what agar and pH (5 or 7) is optimal. Isolation may also be further complicated by the fact that, in most cases, multiple, already known, canine and feline associated gastric NHPHs colonize the stomach of dogs and cats, which may be able to grow better in the culture conditions applied.

In conclusion, the current results obtained through PCR and sequencing analysis and histological examination indicate that cats and dogs may be (co-)infected with gastric *Helicobacter* organisms other than the known gastric NHPHs. Culture and isolation methods should be applied to confirm the presence of these potentially novel *H. pylori*-like organisms and characterize them.

### Supplementary Information

**Additional file 1: PCR and BLAST results per animal and per stomach region.**

**Additional file 2. BLAST details *glmM* PCR 010DA.**

## Data Availability

The datasets used and/or analyzed during the current study are available from the corresponding author on reasonable request.
