# Supplementary material for: Presence of potentially novel Helicobacter pylori-like organisms in gastric samples from cats and dogs
Source: Vet Res. 2023 Oct 17;54:93. doi: 10.1186/s13567-023-01223-4 (PMC10583413; doi:10.1186/s13567-023-01223-4)
Supplement: Supplementary file 1 — Additional file 1: PCR and BLAST results per animal and per stomach region. [file 13567_2023_1223_MOESM1_ESM.docx]

**Additional file 1** **PCR and BLAST results per animal and per stomach region**

| **#** | **Animal species** | **Stomach region** | **Canine/feline associated gastric NHPHs (*16S rRNA* PCR) (animal level)** | ***16S rRNA* BLAST result (sample level)^‡^** | ***H. pylori*-specific *ureAB* PCR (animal level)** | ***ureAB* BLAST result (sample level)^‡^** | ***H. pylori*-specific *glmM* PCR (animal level)** | ***glmM* BLAST result (sample level)^‡^** | **1^st^ PCR of nested *Helicobacter 23S rRNA* PCR (animal level)** | ***23S rRNA* BLAST result (sample level)^‡^** | **Repetition of sequencing for *23S rRNA* BLAST result (sample level)** ^†,^**^‡^** | **2^nd^ PCR of nested *Helicobacter 23S rRNA* PCR (animal level)** |
| --- | --- | --- | --- | --- | --- | --- | --- | --- | --- | --- | --- | --- |
| **1** | Cat | Corpus | 1 | C/FAGH | 1 | *Helicobacter* *pylori* strain G-Mx-2006-46 chromosome | 0 | NA | 1 | *Helicobacter* sp. NHP21005,  *Helicobacter* sp. NHP19-012,  *Helicobacter* sp. NHP19-003 |  | 0 |
|  |  | Antrum |  | C/FAGH |  | NA |  | NA |  | *Helicobacter* sp. NHP21005, *Helicobacter* sp. NHP19-012, *Helicobacter* sp. NHP19-003 |  |  |
| **2** | Cat | Corpus | 1 | C/FAGH | 0 | NA | 0 | NA | 1 | *Helicobacter* *ailurogastricus* ASB7,  *Helicobacter* *bizzozeronii* strain CIII-1 23S ribosomal RNA gene | *Helicobacter* *ailurogastricus* ASB7,  *Helicobacter* *bizzozeronii* strain CIII-1 23S ribosomal RNA gene | 0 |
|  |  | Antrum |  | C/FAGH |  | NA |  | NA |  | *Helicobacter* *heilmannii* ASB1 |  |  |
| **3** | Cat | Corpus | 1 | Query coverage for C/FAGH <95% | 0 | NA | 0 | NA | 0 | NA |  | 0 |
|  |  | Antrum |  | C/FAGH |  | NA |  | NA |  | NA |  |  |
| **4** | Dog | Corpus | 1 | No good quality sequence obtained | 1 | *Helicobacter* *pylori* strain G-Mx-2006-46 chromosome | 0 | NA | 0 | NA |  | 0 |
|  |  | Antrum |  | C/FAGH |  | No good quality sequence obtained |  | NA |  | NA |  |  |
| **5** | Dog | Corpus | 1 | C/FAGH | 0 | Query coverage for *Helicobacter* *pylori* isolate 39 urease subunit A (ureA) and urease subunit B (ureB) genes, partial cds <95% | 0 | No good quality sequence obtained | 1 | *Helicobacter* *pylori* ATCC 49503,  *Helicobacter* *valdiviensis* PAGU 2070 gene for 23S rRNA,  *Helicobacter* sp. PAGU 2000 gene for *23S rRNA* | *Helicobacter* sp. NHP21005, *Helicobacter* *ailurogastricus* ASB7,  *Helicobacter* *heilmannii* ASB1, *Helicobacter* sp. NHP19-012, *Helicobacter* sp. NHP19-003, *Helicobacter* *bizzozeronii* strain CIII-1 23S ribosomal RNA gene,  *Helicobacter* *felis* ATCC 49179,  *Helicobacter* *felis* strain Lee CS5/DS2/CS3/CS1 23S ribosomal RNA | 0 |
|  |  | Antrum |  | C/FAGH |  | NA |  | No good quality sequence obtained |  | No good quality sequence obtained |  |  |
| **6** | Dog | Corpus | 1 | C/FAGH | 0 | NA | 0 | NA | 0 | NA |  | 0 |
|  |  | Antrum |  | C/FAGH |  | NA |  | NA |  | NA |  |  |
| **7** | Cat | Corpus | 1 | C/FAGH | 1 | *Helicobacter* *pylori* strain G-Mx-2006-46 chromosome | 0 | NA | 1 | *Helicobacter* *heilmannii* ASB1 |  | 0 |
|  |  | Antrum |  | C/FAGH |  | *Helicobacter* *pylori* strain HP1352 chromosome |  | NA |  | *Helicobacter* *heilmannii* ASB1 |  |  |
| **8** | Dog | Corpus | 1 | C/FAGH | 0 | NA | 0 | NA | 1 | NA |  | 0 |
|  |  | Antrum |  | C/FAGH |  | NA |  | NA |  | *Helicobacter* sp. NHP21005, *Helicobacter* *ailurogastricus* ASB7,  *Helicobacter* *heilmannii* ASB1,  *Helicobacter* sp. NHP19-012, *Helicobacter* sp. NHP19-003, *Helicobacter* *bizzozeronii* strain CIII-1 23S ribosomal RNA gene,  *Helicobacter* *felis* ATCC 49179,  *Helicobacter* *felis* strain Lee CS5/DS2/CS3/CS1 23S ribosomal RNA |  |  |
| **9** | Cat | Corpus | 0 | NA | 1 | *Helicobacter* *pylori* strain G-Mx-2006-46 chromosome | 0 | NA | 0 | NA |  | 0 |
|  |  | Antrum |  | NA |  | NA |  | NA |  | NA |  |  |
| **10** | Dog | Corpus | 1 | C/FAGH | 1 | *Helicobacter* *pylori* strain HP1352 chromosome | 1 | NA | 1 | *Helicobacter* *heilmannii* ASB1 |  | 0 |
|  |  | Antrum |  | C/FAGH |  | *Helicobacter* *pylori* strain C-Mx-2010-3 chromosome |  | *Helicobacter* *pylori* strain G-Mx-2003-108 chromosome (% identity = 95%) |  | *Helicobacter* sp. NHP21005, *Helicobacter* sp. NHP19-012, *Helicobacter* sp. NHP19-003 |  |  |
| **11** | Cat | Corpus | 1 | C/FAGH | 0 | NA | 0 | NA | 1 | *Helicobacter* *ailurogastricus* ASB7 |  | 0 |
|  |  | Antrum |  | C/FAGH |  | No good quality sequence obtained |  | NA |  | *Helicobacter* sp. NHP21005, *Helicobacter* *ailurogastricus* ASB7,  *Helicobacter* *heilmannii* ASB1,  *Helicobacter* sp. NHP19-012, *Helicobacter* sp. NHP19-003, *Helicobacter* *bizzozeronii* strain CIII-1 23S ribosomal RNA gene,  *Helicobacter* *felis* ATCC 49179,  *Helicobacter* *felis* strain Lee CS5/DS2/CS3/CS1 23S ribosomal RNA |  |  |
| **12** | Cat | Corpus | 1 | C/FAGH | 1 | *Helicobacter* *pylori* isolate 39 urease subunit A (ureA) and urease subunit B (ureB) genes, partial cds | 0 | NA | 1 | *Helicobacter* *pylori* gene for 23S ribosomal RNA, partial sequence, isolate: CLON98 | *Helicobacter* *pylori* gene for 23S ribosomal RNA, partial sequence, isolate: CLON98 | 0 |
|  |  | Antrum |  | NA |  | *Helicobacter* *pylori* strain G-Mx-2006-46 chromosome |  | NA |  | NA |  |  |
| **13** | Cat | Corpus | 1 | C/FAGH | 1 | *Helicobacter* *pylori* strain G-Mx-2006-46 chromosome | 0 | NA | 1 | *Helicobacter* *ailurogastricus* ASB7 |  | 0 |
|  |  | Antrum |  | C/FAGH |  | NA |  | NA |  | *Helicobacter* *heilmannii* ASB1 |  |  |
| **14** | Dog | Corpus | 1 | C/FAGH | 0 | NA | 0 | NA | 1 | NA |  | 0 |
|  |  | Antrum |  | C/FAGH |  | NA |  | NA |  | *Helicobacter* sp. NHP21005, *Helicobacter* sp. NHP19-012, *Helicobacter* sp. NHP19-003 |  |  |
| **15** | Cat | Corpus | 1 | C/FAGH | 0 | NA | 0 | NA | 1 | *Helicobacter* *heilmannii* ASB1 |  | 0 |
|  |  | Antrum |  | C/FAGH |  | NA |  | NA |  | *Helicobacter* *heilmannii* ASB1 |  |  |
| **16** | Cat | Corpus | 0 | No good quality sequence obtained | 1 | NA | 0 | NA | 0 | NA |  | 0 |
|  |  | Antrum |  | NA |  | *Helicobacter* *pylori* strain G-Mx-2006-46 chromosome |  | NA |  | NA |  |  |
| **17** | Dog | Corpus | 1 | C/FAGH | 0 | No sequence similarity found | 0 | NA | 1 | *Helicobacter* *felis* strain Lee DS2 23S ribosomal RNA gene |  | 0 |
|  |  | Antrum |  | C/FAGH |  | NA |  | NA |  | *Helicobacter* *felis* strain Lee DS2 23S ribosomal RNA gene |  |  |
| **18** | Dog | Corpus | 0 | NA | 1 | NA | 0 | NA | 0 | NA |  | 0 |
|  |  | Antrum |  | NA |  | *Helicobacter* *pylori* DNA, complete genome, strain: PMSS1 |  | NA |  | NA |  |  |
| **19** | Cat | Corpus | 1 | C/FAGH | 1 | NA | 0 | NA | 1 | *Helicobacter* *heilmannii* ASB1 |  | 0 |
|  |  | Antrum |  | C/FAGH |  | *Helicobacter* *pylori* strain G-Mx-2006-46 chromosome |  | NA |  | No sequence similarity found |  |  |
| **20** | Cat | Corpus | 1 | C/FAGH | 1 | NA | 0 | NA | 1 | *Helicobacter* sp. NHP21005, *Helicobacter* sp. NHP19-012, *Helicobacter* sp. NHP19-003 |  | 0 |
|  |  | Antrum |  | C/FAGH |  | *Helicobacter* *pylori* strain G-Mx-2006-46 chromosome |  | NA |  | *Helicobacter* sp. NHP21005, *Helicobacter* sp. NHP19-012, *Helicobacter* sp. NHP19-003 |  |  |
| **21** | Cat | Corpus | 1 | C/FAGH | 0 | NA | 0 | NA | 1 | NA |  | 0 |
|  |  | Antrum |  | C/FAGH |  | NA |  | NA |  | *Helicobacter* sp. NHP21005, *Helicobacter* sp. NHP19-012, *Helicobacter* sp. NHP19-003 |  |  |
| **22** | Dog | Corpus | 1 | C/FAGH | 1 | *Helicobacter* *pylori* strain: PMSS1 | 0 | No good quality sequence obtained | 1 | *Helicobacter* sp. NHP21005, *Helicobacter* sp. NHP19-012, *Helicobacter* sp. NHP19-003 |  | 0 |
|  |  | Antrum |  | No good quality sequence obtained |  | NA |  | No good quality sequence obtained |  | *Helicobacter* sp. NHP21005, *Helicobacter* sp. NHP19-012, *Helicobacter* sp. NHP19-003 |  |  |
| **23** | Dog | Corpus | 1 | C/FAGH | 0 | NA | 0 | No good quality sequence obtained | 1 | *Helicobacter* sp. NHP21005, *Helicobacter* sp. NHP19-012, *Helicobacter* sp. NHP19-003, *Helicobacter* *felis* strain Lee CS1 23S ribosomal RNA gene |  | 0 |
|  |  | Antrum |  | C/FAGH |  | NA |  | No good quality sequence obtained |  | *Helicobacter* *heilmannii* ASB1 |  |  |
| **24** | Dog | Corpus | 1 | C/FAGH | 1 | *Helicobacter* *pylori* strain G-Mx-2006-46 chromosome | 0 | No good quality sequence obtained | 1 | *Helicobacter* sp. NHP21005, *Helicobacter* sp. NHP19-012, *Helicobacter* sp. NHP19-003 |  | 0 |
|  |  | Antrum |  | C/FAGH |  | NA |  | No good quality sequence obtained |  | *Helicobacter* sp. NHP21005, *Helicobacter* sp. NHP19-012, *Helicobacter* sp. NHP19-003, *Helicobacter* *felis* strain Lee CS5 23S ribosomal RNA gene |  |  |
| **25** | Dog | Corpus | 1 | C/FAGH | 1 | *Helicobacter* *pylori* strain G-Mx-2006-46 chromosome | 0 | NA | 1 | *Helicobacter* sp. NHP21005, *Helicobacter* sp. NHP19-012, *Helicobacter* sp. NHP19-003, *Helicobacter* *felis* strain Lee CS1 23S ribosomal RNA gene |  | 0 |
|  |  | Antrum |  | C/FAGH |  | *Helicobacter* *pylori* strain G-Mx-2006-46 chromosome |  | NA |  | *Helicobacter* sp. NHP21005, *Helicobacter* sp. NHP19-012, *Helicobacter* sp. NHP19-003, *Helicobacter* *felis* strain Lee CS1 23S ribosomal RNA gene |  |  |
| **26** | Dog | Corpus | 0 | NA | 0 | No good quality sequence obtained | 0 | NA | 0 | NA |  | 0 |
|  |  | Antrum |  | No good quality sequence obtained |  | NA |  | NA |  | No good quality sequence obtained |  |  |
| **27** | Dog | Corpus | 1 | C/FAGH | 0 | NA | 0 | NA | 1 | NA |  | 0 |
|  |  | Antrum |  | C/FAGH |  | NA |  | NA |  | *Helicobacter* sp. NHP21005, *Helicobacter* sp. NHP19-012, *Helicobacter* sp. NHP19-003 |  |  |
| **28** | Dog | Corpus | 1 | C/FAGH | 1 | *Helicobacter* *pylori* isolate 39 urease subunit A (ureA) and urease subunit B (ureB) genes, partial cds | 0 | NA | 1 | *Helicobacter* *heilmannii* ASB1 |  | 0 |
|  |  | Antrum |  | C/FAGH |  | NA |  | NA |  | *Helicobacter* *felis* strain Lee DS2 23S ribosomal RNA |  |  |
| **29** | Cat | Corpus | 1 | C/FAGH | 0 | NA | 0 | NA | 1 | *Helicobacter* *ailurogastricus* ASB7 |  | 0 |
|  |  | Antrum |  | C/FAGH |  | NA |  | NA |  | *Helicobacter* sp. NHP21005, *Helicobacter* *ailurogastricus* ASB7,  *Helicobacter* *heilmannii* ASB1,  *Helicobacter* sp. NHP19-012, *Helicobacter* sp. NHP19-003, *Helicobacter* *bizzozeronii* strain CIII-1 23S ribosomal RNA gene,  *Helicobacter* *felis* ATCC 49179,  *Helicobacter* *felis* strain Lee CS5/DS2/CS3/CS1 23S ribosomal RNA |  |  |
| **30** | Cat | Corpus | 1 | C/FAGH | 0 | NA | 0 | NA | 0 | NA |  | 0 |
|  |  | Antrum |  | No good quality sequence obtained |  | NA |  | NA |  | NA |  |  |
| **31** | Dog | Corpus | 1 | C/FAGH | 0 | NA | 0 | NA | 1 | *Helicobacter* sp. NHP21005, *Helicobacter* sp. NHP19-012, *Helicobacter* sp. NHP19-003, *Helicobacter* *felis* strain Lee CS1 23S ribosomal RNA gene |  | 0 |
|  |  | Antrum |  | C/FAGH |  | NA |  | NA |  | *Helicobacter* sp. NHP21005, *Helicobacter* *ailurogastricus* ASB7,  *Helicobacter* *heilmannii* ASB1,  *Helicobacter* sp. NHP19-012, *Helicobacter* sp. NHP19-003, *Helicobacter* *bizzozeronii* strain CIII-1 23S ribosomal RNA gene,  *Helicobacter* *felis* ATCC 49179,  *Helicobacter* *felis* strain Lee CS5/DS2/CS3/CS1 23S ribosomal RNA |  |  |
| **32** | Dog | Corpus | 0 | NA | 1 | NA | 0 | NA | 1 | NA |  | 0 |
|  |  | Antrum |  | NA |  | *Helicobacter* *pylori* strain G-Mx-2006-46 chromosome |  | NA |  | *Helicobacter* sp. NHP21005, *Helicobacter* sp. NHP19-012, *Helicobacter* sp. NHP19-003 |  |  |
| **33** | Cat | Corpus | 1 | C/FAGH | 0 | NA | 0 | NA | 1 | *Helicobacter* sp. NHP21005, *Helicobacter* sp. NHP19-012, *Helicobacter* sp. NHP19-003 |  | 0 |
|  |  | Antrum |  | C/FAGH |  | NA |  | NA |  | *Helicobacter* sp. NHP21005, *Helicobacter* sp. NHP19-012, *Helicobacter* sp. NHP19-003 |  |  |
| **34** | Cat | Corpus | 1 | C/FAGH | 1 | *Helicobacter* *pylori* strain G-Mx-2006-46 chromosome | 0 | NA | 1 | *Helicobacter* *ailurogastricus* ASB7,  *Helicobacter* *heilmannii* ASB1 |  | 0 |
|  |  | Antrum |  | C/FAGH |  | No good quality sequence obtained |  | NA |  | No good quality sequence obtained |  |  |
| **35** | Dog | Corpus | 1 | C/FAGH | 0 | % identity for *Helicobacter* *pylori* strain G-Mx-2006-46 chromosome <<96% | 0 | NA | 1 | *Helicobacter* sp. NHP21005, *Helicobacter* sp. NHP19-012, *Helicobacter* sp. NHP19-003 |  | 0 |
|  |  | Antrum |  | C/FAGH |  | NA |  | NA |  | *Helicobacter* sp. NHP21005, *Helicobacter* sp. NHP19-012, *Helicobacter* sp. NHP19-003 |  |  |
| **36** | Cat | Corpus | 1 | C/FAGH | 0 | No good quality sequence obtained | 0 | NA | 0 | NA |  | 0 |
|  |  | Antrum |  | No good quality sequence obtained |  | NA |  | NA |  | NA |  |  |
| **37** | Dog | Corpus | 1 | No good quality sequence obtained | 1 | NA | 0 | NA | 1 | *Helicobacter* sp. NHP21005, *Helicobacter* sp. NHP19-012, *Helicobacter* sp. NHP19-003 |  | 0 |
|  |  | Antrum |  | C/FAGH |  | *Helicobacter* *pylori* isolate 39 urease subunit A (ureA) and urease subunit B (ureB) genes, partial cds |  | NA |  | *Helicobacter* sp. NHP21005, *Helicobacter* sp. NHP19-012, *Helicobacter* sp. NHP19-003 |  |  |
| **38** | Dog | Corpus | 1 | C/FAGH | 1 | *Helicobacter* *pylori* strain G-Mx-2006-46 chromosome | 0 | NA | 1 | *Helicobacter* *pylori* ATCC 49503,  *Helicobacter* *valdiviensis* PAGU 2070 gene for *23S rRNA*,  *Helicobacter* sp. PAGU 2000 gene for *23S rRNA* | *Helicobacter* sp. NHP21005, *Helicobacter* *ailurogastricus* ASB7,  *Helicobacter* *heilmannii* ASB1, *Helicobacter* sp. NHP19-012, *Helicobacter* sp. NHP19-003, *Helicobacter* *bizzozeronii* strain CIII-1 23S ribosomal RNA gene,  *Helicobacter* *felis* ATCC 49179,  *Helicobacter* *felis* strain Lee CS5/DS2/CS3/CS1 23S ribosomal RNA | 0 |
|  |  | Antrum |  | C/FAGH |  | *Helicobacter* *pylori* strain G-Mx-2006-46 chromosome |  | NA |  | *Helicobacter* sp. NHP21005, *Helicobacter* sp. NHP19-012, *Helicobacter* sp. NHP19-003 |  |  |
| **39** | Dog | Corpus | 1 | C/FAGH | 0 | NA | 0 | NA | 1 | *Helicobacter* *heilmannii* ASB1 |  | 0 |
|  |  | Antrum |  | C/FAGH |  | NA |  | NA |  | NA |  |  |
| **40** | Dog | Corpus | 0 | NA | 0 | NA | 0 | NA | 0 | NA |  | 0 |
|  |  | Antrum |  | NA |  | NA |  | No good quality sequence obtained |  | NA |  |  |
| **41** | Dog | Corpus | 1 | C/FAGH | 0 | No good quality sequence obtained | 0 | NA | 1 | *Helicobacter* sp. NHP21005, *Helicobacter* sp. NHP19-012, *Helicobacter* sp. NHP19-003 (% identity = 95.5%) |  | 0 |
|  |  | Antrum |  | No good quality sequence obtained |  | % identity for *Helicobacter* *pylori* strain G-Mx-2005-337 chromosome <<96% |  | NA |  | *Helicobacter* sp. NHP21005, *Helicobacter* sp. NHP19-012, *Helicobacter* sp. NHP19-003 |  |  |
| **42** | Cat | Corpus | 1 | C/FAGH | 0 | No good quality sequence obtained | 0 | NA | 1 | *Helicobacter* sp. NHP21005, *Helicobacter* sp. NHP19-012, *Helicobacter* sp. NHP19-003 |  | 0 |
|  |  | Antrum |  | C/FAGH |  | NA |  | NA |  | No good quality sequence obtained |  |  |
| **43** | Dog | Corpus | 1 | C/FAGH | 1 | *Helicobacter* *pylori* isolate 39 urease subunit A (ureA) and urease subunit B (ureB) genes, partial cds | 0 | NA | 1 | *Helicobacter* sp. NHP21005, *Helicobacter* sp. NHP19-012, *Helicobacter* sp. NHP19-003 |  | 0 |
|  |  | Antrum |  | C/FAGH |  | NA |  | NA |  | *Helicobacter* sp. NHP21005, *Helicobacter* sp. NHP19-012, *Helicobacter* sp. NHP19-003 |  |  |
| **44** | Dog | Corpus | 1 | C/FAGH | 1 | *Helicobacter* *pylori* strain G-Mx-2006-46 chromosome | 0 | NA | 1 | *Helicobacter* sp. NHP21005, *Helicobacter* sp. NHP19-012, *Helicobacter* sp. NHP19-003, *Helicobacter* *felis* strain Lee CS1 23S ribosomal RNA gene |  | 0 |
|  |  | Antrum |  | C/FAGH |  | NA |  | No good quality sequence obtained |  | *Helicobacter* *ailurogastricus* ASB7 DNA |  |  |
| **45** | Dog | Corpus | 1 | C/FAGH | 1 | *Helicobacter* *pylori* strain C-Mx-2010-3 chromosome | 0 | NA | 1 | *Helicobacter* *heilmannii* ASB1 |  | 0 |
|  |  | Antrum |  | C/FAGH |  | *Helicobacter* *pylori* strain HP1352 chromosome |  | NA |  | *Helicobacter* *heilmannii* ASB1 |  |  |
| **46** | Dog | Corpus | 0 | No good quality sequence obtained | 0 | No good quality sequence obtained | 0 | NA | 0 | NA |  | 0 |
|  |  | Antrum |  | No good quality sequence obtained |  | NA |  | NA |  | NA |  |  |
| **47** | Cat | Corpus | 0 | No good quality sequence obtained | 0 | NA | 0 | NA | 1 | *Helicobacter* *ailurogastricus* ASB7 |  | 0 |
|  |  | Antrum |  | No good quality sequence obtained |  | NA |  | NA |  | NA |  |  |

**^‡^** The *Helicobacter* species/strains included in this table are the top BLAST results showing highest % identity; ^†^ Sequencing of the amplicon was repeated for the samples for which this column is completed, because the initially obtained sequence was significantly shorter compared to the expected amplicon size; 1 = at least one sample of the animal was positive in the corresponding PCR assay and the result was confirmed by sequencing; 0 = none of the samples of the animal were positive in the corresponding PCR assay; C/FAGH = group of canine/feline associated gastric *Helicobacter* species; NA = not applicable (because no PCR amplicon was obtained, or the PCR result was proven false positive upon sequencing); << = far lower than
