# Supplementary material for: Presence of potentially novel Helicobacter pylori-like organisms in gastric samples from cats and dogs
Source: Vet Res. 2023 Oct 17;54:93. doi: 10.1186/s13567-023-01223-4 (PMC10583413; doi:10.1186/s13567-023-01223-4)
Supplement: Supplementary file 2 — Additional file 2. BLAST details glmM PCR 010DA. [file 13567_2023_1223_MOESM2_ESM.pdf]

Description	Scientific Name	Max Score	Total Score	Query Cover	E value	Per. Ident	Acc. Len	Accession
Helicobacter pylori strain G-Mx-2003-108 chromosome	Helicobacter pylori	97.1	97.1	100%	2e-16	95.16%	1667946	CP032044.1
Helicobacter pylori strain G-Mx-2006-152 chromosome	Helicobacter pylori	97.1	97.1	100%	2e-16	95.16%	1613306	CP032040.1
Helicobacter pylori DNA, complete genome, strain: PMSS1	Helicobacter pylori	97.1	97.1	95%	2e-16	96.61%	1603093	AP017633.1
Helicobacter pylori strain FDAARGOS_300 chromosome, complete genome	Helicobacter pylori	97.1	97.1	100%	2e-16	95.16%	1645738	CP027404.1
Helicobacter pylori SS1, complete genome	Helicobacter pylori SS1	97.1	97.1	100%	2e-16	95.16%	1619098	CP009259.1
Helicobacter pylori strain PMSS1 complete genome	Helicobacter pylori PMSS1	97.1	97.1	100%	2e-16	95.16%	1618480	CP018823.1
Helicobacter pylori J99, complete genome	Helicobacter pylori J99	97.1	97.1	100%	2e-16	95.16%	1698366	CP011330.1
Helicobacter pylori strain oh1 chromosome	Helicobacter pylori	97.1	97.1	100%	2e-16	95.16%	1600023	CP109885.1
Helicobacter pylori strain J99 derivative isolate C2 adapted chromosome, complete genome	Helicobacter pylori	97.1	97.1	100%	2e-16	95.16%	1653204	CP089284.1
Helicobacter pylori strain J99 derivative isolate D1 chromosome, complete genome	Helicobacter pylori	97.1	97.1	100%	2e-16	95.16%	1685094	CP089283.1
Helicobacter pylori strain LIM-009 chromosome, complete genome	Helicobacter pylori	97.1	97.1	100%	2e-16	95.16%	1618010	CP051534.1
Helicobacter pylori strain 603T phosphoglucosamine mutase (glmM) gene, partial cds	Helicobacter pylori	97.1	97.1	100%	2e-16	95.16%	555	JN390578.1
Helicobacter pylori strain 600T phosphoglucosamine mutase (glmM) gene, partial cds	Helicobacter pylori	97.1	97.1	100%	2e-16	95.16%	555	JN390576.1
Helicobacter pylori strain 595T phosphoglucosamine mutase (glmM) gene, partial cds	Helicobacter pylori	97.1	97.1	100%	2e-16	95.16%	555	JN390574.1
Helicobacter pylori strain 590T phosphoglucosamine mutase (glmM) gene, partial cds	Helicobacter pylori	97.1	97.1	100%	2e-16	95.16%	555	JN390573.1
Helicobacter pylori strain 370H phosphoglucosamine mutase (glmM) gene, partial cds	Helicobacter pylori	97.1	97.1	100%	2e-16	95.16%	555	JN390551.1
Helicobacter pylori Gambia94/24, complete genome	Helicobacter pylori Gambia94/24	97.1	97.1	100%	2e-16	95.16%	1709911	CP002332.1
Helicobacter pylori strain PP37A1 phosphoglucosamine	Helicobacter pylori	97.1	97.1	100%	2e-16	95.16%	555	GU045999.1

Description	Scientific Name	Max Score	Total Score	Query Cover	E value	Per. Ident	Acc. Len	Accession
mutase (glmM) gene, partial cds								
Helicobacter pylori strain S23 phosphoglucosamine mutase (glmM) gene, partial cds	Helicobacter pylori	97.1	97.1	100%	2e-16	95.16%	555	GU045994.1
Helicobacter pylori strain PS148C1 phosphoglucosamine mutase (glmM) gene, partial cds	Helicobacter pylori	97.1	97.1	100%	2e-16	95.16%	555	GU045991.1
Helicobacter pylori J99, complete genome	Helicobacter pylori J99	97.1	97.1	100%	2e-16	95.16%	1643831	AE001439.1
Helicobacter pylori strain JF80 phosphoglucosamine mutase (glmM) gene, partial cds	Helicobacter pylori	97.1	97.1	100%	2e-16	95.16%	591	AY152929.1
Helicobacter pylori strain PP39A1 phosphoglucosamine mutase (glmM) gene, partial cds	Helicobacter pylori	97.1	97.1	100%	2e-16	95.16%	591	AY152913.1
Helicobacter pylori strain PS56C phosphoglucosamine mutase (glmM) gene, partial cds	Helicobacter pylori	97.1	97.1	100%	2e-16	95.16%	591	AY152896.1
Helicobacter pylori strain S57 phosphoglucosamine mutase (glmM) gene, partial cds	Helicobacter pylori	97.1	97.1	100%	2e-16	95.16%	591	AY152893.1
Helicobacter pylori strain S55 phosphoglucosamine mutase (glmM) gene, partial cds	Helicobacter pylori	97.1	97.1	100%	2e-16	95.16%	591	AY152892.1
Helicobacter pylori isolate HE93/10_v1 genome assembly, chromosome: I	Helicobacter pylori	93.5	93.5	88%	2e-15	98.15%	1682800	LT838273.1
Helicobacter pylori isolate BCM-300 genome assembly, chromosome: I	Helicobacter pylori	93.5	93.5	88%	2e-15	98.15%	1667883	LT837687.1
Helicobacter pylori isolate HE142/09 genome assembly, chromosome: I	Helicobacter pylori	93.5	93.5	88%	2e-15	98.15%	1667936	LT635478.1
Helicobacter pylori isolate HE147/09 genome assembly, chromosome: I	Helicobacter pylori	93.5	93.5	88%	2e-15	98.15%	1667712	LT635477.1
Helicobacter pylori isolate HE134/09 genome assembly, chromosome: I	Helicobacter pylori	93.5	93.5	88%	2e-15	98.15%	1667858	LT635476.1
Helicobacter pylori isolate HE171/09 genome assembly, chromosome: I	Helicobacter pylori	93.5	93.5	88%	2e-15	98.15%	1667625	LT635474.1
Helicobacter pylori isolate HE136/09 genome assembly, chromosome: I	Helicobacter pylori	93.5	93.5	88%	2e-15	98.15%	1667978	LT635473.1
Helicobacter pylori isolate HE170/09 genome assembly, chromosome: I	Helicobacter pylori	93.5	93.5	88%	2e-15	98.15%	1670321	LT635472.1

Description	Scientific Name	Max Score	Total Score	Query Cover	E value	Per. Ident	Acc. Len	Accession
Helicobacter pylori isolate HE141/09 genome assembly, chromosome: I	Helicobacter pylori	93.5	93.5	88%	2e-15	98.15%	1670384	LT635471.1
Helicobacter pylori isolate HE178/09 genome assembly, chromosome: I	Helicobacter pylori	93.5	93.5	88%	2e-15	98.15%	1667894	LT635460.1
Helicobacter pylori isolate HE132/09 genome assembly, chromosome: I	Helicobacter pylori	93.5	93.5	88%	2e-15	98.15%	1668174	LT635459.1
Helicobacter pylori isolate HE143/09 genome assembly, chromosome: I	Helicobacter pylori	93.5	93.5	88%	2e-15	98.15%	1667804	LT635458.1
Helicobacter pylori isolate HE101/09 genome assembly, chromosome: I	Helicobacter pylori	93.5	93.5	88%	2e-15	98.15%	1667821	LT635456.1
Helicobacter pylori strain G-Mx-2005-108 chromosome	Helicobacter pylori	91.6	91.6	100%	8e-15	93.55%	1654849	CP032042.1
Helicobacter pylori strain G-Mx-2011-124 chromosome	Helicobacter pylori	91.6	91.6	100%	8e-15	93.55%	1661266	CP032038.1
Helicobacter pylori strain C-Mx-2011-171 chromosome	Helicobacter pylori	91.6	91.6	100%	8e-15	93.55%	1645502	CP032033.1
Helicobacter pylori strain C-Mx-2010-8 chromosome	Helicobacter pylori	91.6	91.6	100%	8e-15	93.55%	1682275	CP032027.1
Helicobacter pylori strain B147 chromosome, complete genome	Helicobacter pylori	91.6	91.6	100%	8e-15	93.55%	1631093	CP024946.1
Helicobacter pylori strain B140 chromosome, complete genome	Helicobacter pylori	91.6	91.6	100%	8e-15	93.55%	1726836	CP024948.1
Helicobacter pylori strain 24-A-EK1 chromosome, complete genome	Helicobacter pylori	91.6	91.6	100%	8e-15	93.55%	1659500	CP032907.1
Helicobacter pylori strain 478-A-EK1 chromosome, complete genome	Helicobacter pylori	91.6	91.6	100%	8e-15	93.55%	1628623	CP032899.1
Helicobacter pylori strain HP14039 chromosome, complete genome	Helicobacter pylori	91.6	91.6	100%	8e-15	93.55%	1678260	CP034147.1
Helicobacter pylori DNA, complete genome, strain: ATCC43504	Helicobacter pylori	91.6	91.6	100%	8e-15	93.55%	1680829	AP017632.1
Helicobacter pylori strain NCTC11637 genome assembly, chromosome: 1	Helicobacter pylori NCTC 11637 = CCUG 17874 = ATCC 43504 = JCM 12093	91.6	91.6	100%	8e-15	93.55%	1680937	LS483488.1
Helicobacter pylori strain FDAARGOS_298 chromosome, complete genome	Helicobacter pylori	91.6	91.6	100%	8e-15	93.55%	1680898	CP028325.1
Helicobacter pylori strain 444A6 chromosome, complete genome	Helicobacter pylori	91.6	91.6	100%	8e-15	93.55%	1621210	CP117024.1
Helicobacter pylori strain K262	Helicobacter pylori	91.6	91.6	100%	8e-15	93.55%	1645008	CP091772.1

Description	Scientific Name	Max Score	Total Score	Query Cover	E value	Per. Ident	Acc. Len	Accession
chromosome, complete genome								
Helicobacter pylori strain MT5118 chromosome, complete genome	Helicobacter pylori	91.6	91.6	100%	8e-15	93.55%	1628076	CP071975.1
Helicobacter pylori strain MT5125 chromosome, complete genome	Helicobacter pylori	91.6	91.6	100%	8e-15	93.55%	1685803	CP071978.1
Helicobacter pylori strain MT5111 chromosome, complete genome	Helicobacter pylori	91.6	91.6	100%	8e-15	93.55%	1668381	CP071981.1
Helicobacter pylori strain MT5135 chromosome, complete genome	Helicobacter pylori	91.6	91.6	100%	8e-15	93.55%	1615199	CP071982.1
Helicobacter pylori strain MT5114 chromosome, complete genome	Helicobacter pylori	91.6	91.6	100%	8e-15	93.55%	1690568	CP071984.1
Helicobacter pylori strain MT5119 chromosome, complete genome	Helicobacter pylori	91.6	91.6	100%	8e-15	93.55%	1622324	CP071985.1
Helicobacter pylori strain LIM-001 chromosome, complete genome	Helicobacter pylori	91.6	91.6	100%	8e-15	93.55%	1638284	CP051541.1
Helicobacter pylori strain LIM-010 chromosome, complete genome	Helicobacter pylori	91.6	91.6	100%	8e-15	93.55%	1745797	CP051533.1
Helicobacter pylori strain 584T phosphoglucosamine mutase (glmM) gene, partial cds	Helicobacter pylori	91.6	91.6	100%	8e-15	93.55%	555	JN390572.1
Helicobacter pylori strain 2161N phosphoglucosamine mutase (glmM) gene, partial cds	Helicobacter pylori	91.6	91.6	100%	8e-15	93.55%	555	JN390567.1
Helicobacter pylori strain 2133N phosphoglucosamine mutase (glmM) gene, partial cds	Helicobacter pylori	91.6	91.6	100%	8e-15	93.55%	555	JN390566.1
Helicobacter pylori HpKE21 DNA, complete genome	Helicobacter pylori	91.6	91.6	100%	8e-15	93.55%	1648327	AP023320.1
Helicobacter pylori SJM180, complete genome	Helicobacter pylori SJM180	91.6	91.6	100%	8e-15	93.55%	1658051	CP002073.1
Helicobacter pylori strain PP2B4 phosphoglucosamine mutase (glmM) gene, partial cds	Helicobacter pylori	91.6	91.6	100%	8e-15	93.55%	555	GU046000.1
Helicobacter pylori B38 complete genome, strain B38	Helicobacter pylori B38	91.6	91.6	100%	8e-15	93.55%	1576758	FM991728.1
Helicobacter pylori strain PP23A1 phosphoglucosamine mutase (glmM) gene, partial cds	Helicobacter pylori	91.6	91.6	100%	8e-15	93.55%	591	AY152914.1
Helicobacter pylori strain PP22B2 phosphoglucosamine mutase (glmM) gene, partial cds	Helicobacter pylori	91.6	91.6	100%	8e-15	93.55%	591	AY152911.1

Description	Scientific Name	Max Score	Total Score	Query Cover	E value	Per. Ident	Acc. Len	Accession
Helicobacter pylori strain PS184A phosphoglucosamine mutase (glmM) gene, partial cds	Helicobacter pylori	91.6	91.6	100%	8e-15	93.55%	591	AY152903.1
Helicobacter pylori strain S49 phosphoglucosamine mutase (glmM) gene, partial cds	Helicobacter pylori	91.6	91.6	100%	8e-15	93.55%	591	AY152890.1
Helicobacter pylori strain GCT 43 chromosome, complete genome	Helicobacter pylori	87.9	87.9	93%	1e-13	94.74%	1642466	CP048600.1
Helicobacter pylori strain C-Mx-2010-97 chromosome	Helicobacter pylori	87.9	87.9	88%	1e-13	96.30%	1624459	CP032036.1
Helicobacter pylori strain C-Mx-2010-3 chromosome	Helicobacter pylori	87.9	87.9	93%	1e-13	94.74%	1642343	CP032023.1
Helicobacter pylori strain C-Mx-2010-5 chromosome	Helicobacter pylori	87.9	87.9	88%	1e-13	96.30%	1645840	CP032022.1
Helicobacter pylori strain MT5105 chromosome, complete genome	Helicobacter pylori	87.9	87.9	88%	1e-13	96.30%	1667928	CP071977.1
Helicobacter pylori strain MT5124 chromosome, complete genome	Helicobacter pylori	87.9	87.9	88%	1e-13	96.30%	1661144	CP071986.1
Helicobacter pylori strain MT5136 chromosome, complete genome	Helicobacter pylori	87.9	87.9	88%	1e-13	96.30%	1660636	CP071987.1
Helicobacter pylori strain PUNO-003 chromosome, complete genome	Helicobacter pylori	87.9	87.9	88%	1e-13	96.30%	1630535	CP051500.1
Helicobacter pylori strain PUNO-009 chromosome, complete genome	Helicobacter pylori	87.9	87.9	88%	1e-13	96.30%	1629514	CP051495.1
Helicobacter pylori 2017, complete genome	Helicobacter pylori 2017	87.9	87.9	88%	1e-13	96.30%	1548238	CP002571.1
Helicobacter pylori 2018, complete genome	Helicobacter pylori 2018	87.9	87.9	88%	1e-13	96.30%	1562832	CP002572.1
Helicobacter pylori 908, complete genome	Helicobacter pylori 908	87.9	87.9	88%	1e-13	96.30%	1549666	CP002184.1
Helicobacter pylori strain PS184A1 phosphoglucosamine mutase (glmM) gene, partial cds	Helicobacter pylori	87.9	87.9	93%	1e-13	94.74%	555	GU045992.1
Helicobacter pylori strain PP25A1 phosphoglucosamine mutase (glmM) gene, partial cds	Helicobacter pylori	87.9	87.9	93%	1e-13	94.74%	591	AY152915.1
Helicobacter pylori strain PP01A1 phosphoglucosamine mutase (glmM) gene, partial cds	Helicobacter pylori	87.9	87.9	93%	1e-13	94.74%	591	AY152909.1
Helicobacter pylori strain PS158A1 phosphoglucosamine mutase (glmM) gene, partial cds	Helicobacter pylori	87.9	87.9	93%	1e-13	94.74%	591	AY152900.1

Description	Scientific Name	Max Score	Total Score	Query Cover	E value	Per. Ident	Acc. Len	Accession
Helicobacter pylori strain S22 phosphoglucosamine mutase (glmM) gene, partial cds	Helicobacter pylori	87.9	87.9	93%	1e-13	94.74%	591	**AY152885.1**
H.pylori urease (ureA, ureB, ureC, ureD) genes, complete cds	Helicobacter pylori	87.9	87.9	93%	1e-13	94.74%	5100	**M60398.1**

Graphic Summary

Distribution of the top 90 Blast Hits on 90 subject sequences

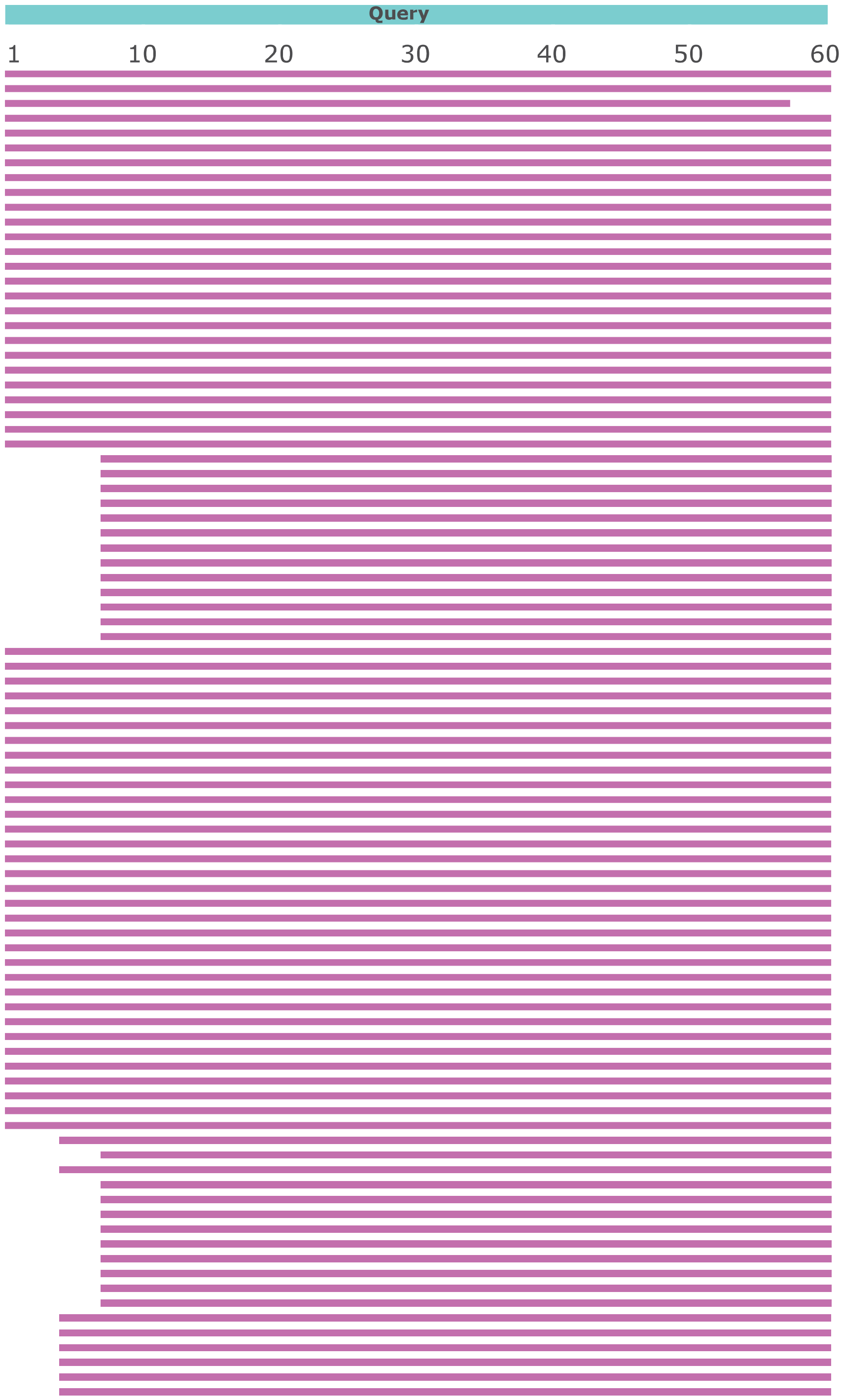

Alignments

Alignment view Pairwise CDS feature Restore defaults

Helicobacter pylori strain G-Mx-2003-108 chromosome

Sequence ID: **CP032044.1** Length: 1667946 Number of Matches: 1

Range 1: 1625821 to 1625882

Score	Expect	Identities	Gaps	Strand	Frame
97.1 bits(52)	2e-16()	59/62(95%)	2/62(3%)	Plus/Plus	

```
Query  1        GTGATCC-AATAGGGCCTATGCCTACCCCTGCGATCGCATTTTTAACCGAA-ACATGCGC  58
                ||||||| |||||||||||||||||||||||||||||| |||||||||||| ||||||||
Sbjct  1625821  GTGATCCAAATAGGGCCTATGCCTACCCCTGCGATCGCTTTTTTAACCGAAGACATGCGC  1625880

Query  59       TG  60
                ||
Sbjct  1625881  TG  1625882
```

Helicobacter pylori strain G-Mx-2006-152 chromosome

Sequence ID: **CP032040.1** Length: 1613306 Number of Matches: 1

Range 1: 820933 to 820994

Score	Expect	Identities	Gaps	Strand	Frame
97.1 bits(52)	2e-16()	59/62(95%)	2/62(3%)	Plus/Plus	

```
Query  1       GTGATCC-AATAGGGCCTATGCCTACCCCTGCGATCGCATTTTTAACCGAA-ACATGCGC  58
               ||||||| |||||||||||||||||||||||||||||| |||||||||||| ||||||||
Sbjct  820933  GTGATCCAAATAGGGCCTATGCCTACCCCTGCGATCGCGTTTTTAACCGAAGACATGCGC  820992

Query  59      TG  60
               ||
Sbjct  820993  TG  820994
```

Helicobacter pylori DNA, complete genome, strain: PMSS1

Sequence ID: **AP017633.1** Length: 1603093 Number of Matches: 1

Range 1: 1447857 to 1447915

Score	Expect	Identities	Gaps	Strand	Frame
97.1 bits(52)	2e-16()	57/59(97%)	2/59(3%)	Plus/Plus	

```
Query  1        GTGATCC-AATAGGGCCTATGCCTACCCCTGCGATCGCATTTTTAACCGAA-ACATGCG  57
                ||||||| ||||||||||||||||||||||||||||||||||||||||||| |||||||
Sbjct  1447857  GTGATCCAAATAGGGCCTATGCCTACCCCTGCGATCGCATTTTTAACCGAAGACATGCG  1447915
```

Helicobacter pylori strain FDAARGOS_300 chromosome, complete genome

Sequence ID: **CP027404.1** Length: 1645738 Number of Matches: 1

Range 1: 780799 to 780860

Score	Expect	Identities	Gaps	Strand	Frame
97.1 bits(52)	2e-16()	59/62(95%)	2/62(3%)	Plus/Minus	

```
Query  1       GTGATCC-AATAGGGCCTATGCCTACCCCTGCGATCGCATTTTTAACCGAA-ACATGCGC  58
               ||||||| |||||||||||||||||||||||||||||| |||||||||||| ||||||||
Sbjct  780860  GTGATCCAAATAGGGCCTATGCCTACCCCTGCGATCGCTTTTTTAACCGAAGACATGCGC  780801

Query  59      TG  60
               ||
Sbjct  780800  TG  780799
```

Helicobacter pylori SS1, complete genome

Sequence ID: **CP009259.1** Length: 1619098 Number of Matches: 1

Range 1: 73410 to 73471

Score	Expect	Identities	Gaps	Strand	Frame
97.1 bits(52)	2e-16()	59/62(95%)	2/62(3%)	Plus/Minus	

```
Query  1      GTGATCC-AATAGGGCCTATGCCTACCCCTGCGATCGCATTTTTAACCGAA-ACATGCGC  58
              ||||||| ||||||||||||||||||||||||||||||||||||||||||| ||||||| 
Sbjct  73471  GTGATCCAAATAGGGCCTATGCCTACCCCTGCGATCGCATTTTTAACCGAAGACATGCGT  73412

Query  59     TG  60
              ||
Sbjct  73411  TG  73410
```

Helicobacter pylori strain PMSS1 complete genome

Sequence ID: **CP018823.1** Length: 1618480 Number of Matches: 1

Range 1: 73411 to 73472

Score	Expect	Identities	Gaps	Strand	Frame
97.1 bits(52)	2e-16()	59/62(95%)	2/62(3%)	Plus/Minus	

```
Query  1      GTGATCC-AATAGGGCCTATGCCTACCCCTGCGATCGCATTTTTAACCGAA-ACATGCGC  58
              ||||||| ||||||||||||||||||||||||||||||||||||||||||| |||||||
Sbjct  73472  GTGATCCAAATAGGGCCTATGCCTACCCCTGCGATCGCATTTTTAACCGAAGACATGCGT  73413

Query  59     TG  60
              ||
Sbjct  73412  TG  73411
```

Helicobacter pylori J99, complete genome

Sequence ID: **CP011330.1** Length: 1698366 Number of Matches: 1

Range 1: 978637 to 978698

Score	Expect	Identities	Gaps	Strand	Frame
97.1 bits(52)	2e-16()	59/62(95%)	2/62(3%)	Plus/Plus	

```
Query  1       GTGATCC-AATAGGGCCTATGCCTACCCCTGCGATCGCATTTTTAACCGAA-ACATGCGC  58
               ||||||| |||||||||||||||||||||||||||||| |||||||||||| ||||||||
Sbjct  978637  GTGATCCAAATAGGGCCTATGCCTACCCCTGCGATCGCTTTTTTAACCGAAGACATGCGC  978696

Query  59      TG  60
               ||
Sbjct  978697  TG  978698
```

Helicobacter pylori strain oh1 chromosome

Sequence ID: **CP109885.1** Length: 1600023 Number of Matches: 1

Range 1: 654841 to 654902

Score	Expect	Identities	Gaps	Strand	Frame
97.1 bits(52)	2e-16()	59/62(95%)	2/62(3%)	Plus/Minus	

```
Query  1       GTGATCC-AATAGGGCCTATGCCTACCCCTGCGATCGCATTTTTAACCGAA-ACATGCGC  58
               ||||||| ||||||||||||||||||||||||||||||||||||||||||| |||||||
Sbjct  654902  GTGATCCAAATAGGGCCTATGCCTACCCCTGCGATCGCATTTTTAACCGAAGACATGCGT  654843

Query  59      TG  60
               ||
Sbjct  654842  TG  654841
```

Helicobacter pylori strain J99 derivative isolate C2 adapted chromosome, complete genome

Sequence ID: **CP089284.1** Length: 1653204 Number of Matches: 1

Range 1: 1363293 to 1363354

Score	Expect	Identities	Gaps	Strand	Frame
97.1 bits(52)	2e-16()	59/62(95%)	2/62(3%)	Plus/Plus	

```
Query  1        GTGATCC-AATAGGGCCTATGCCTACCCCTGCGATCGCATTTTTAACCGAA-ACATGCGC  58
                ||||||| |||||||||||||||||||||||||||||| |||||||||||| ||||||||
Sbjct  1363293  GTGATCCAAATAGGGCCTATGCCTACCCCTGCGATCGCTTTTTTAACCGAAGACATGCGC  1363352

Query  59       TG  60
                ||
Sbjct  1363353  TG  1363354
```

Helicobacter pylori strain J99 derivative isolate D1 chromosome, complete genome

Sequence ID: **CP089283.1** Length: 1685094 Number of Matches: 1

Range 1: 1106831 to 1106892

Score	Expect	Identities	Gaps	Strand	Frame
97.1 bits(52)	2e-16()	59/62(95%)	2/62(3%)	Plus/Minus	

```
Query  1        GTGATCC-AATAGGGCCTATGCCTACCCCTGCGATCGCATTTTTAACCGAA-ACATGCGC  58
                ||||||| |||||||||||||||||||||||||||||| |||||||||||| ||||||||
Sbjct  1106892  GTGATCCAAATAGGGCCTATGCCTACCCCTGCGATCGCTTTTTTAACCGAAGACATGCGC  1106833

Query  59       TG  60
                ||
Sbjct  1106832  TG  1106831
```

Helicobacter pylori strain LIM-009 chromosome, complete genome

Sequence ID: **CP051534.1** Length: 1618010 Number of Matches: 1

Range 1: 75738 to 75799

Score	Expect	Identities	Gaps	Strand	Frame
97.1 bits(52)	2e-16()	59/62(95%)	2/62(3%)	Plus/Minus	

```
Query  1      GTGATCC-AATAGGGCCTATGCCTACCCCTGCGATCGCATTTTTAACCGAA-ACATGCGC  58
              ||||||| |||||||||||||||||||||||||||||| |||||||||||| ||||||||
Sbjct  75799  GTGATCCAAATAGGGCCTATGCCTACCCCTGCGATCGCGTTTTTAACCGAAGACATGCGC  75740

Query  59     TG  60
              ||
Sbjct  75739  TG  75738
```

Helicobacter pylori strain 603T phosphoglucosamine mutase (glmM) gene, partial cds

Sequence ID: **JN390578.1** Length: 555 Number of Matches: 1

Range 1: 94 to 155

Score	Expect	Identities	Gaps	Strand	Frame
97.1 bits(52)	2e-16()	59/62(95%)	2/62(3%)	Plus/Plus	

```
Query  1    GTGATCC-AATAGGGCCTATGCCTACCCCTGCGATCGCATTTTTAACCGAA-ACATGCGC  58
            ||||||| |||||||||||||||||||||||||||||| |||||||||||| ||||||||
Sbjct  94   GTGATCCAAATAGGGCCTATGCCTACCCCTGCGATCGCTTTTTTAACCGAAGACATGCGC  153

Query  59   TG  60
            ||
Sbjct  154  TG  155
```

Helicobacter pylori strain 600T phosphoglucosamine mutase (glmM) gene, partial cds

Sequence ID: **JN390576.1** Length: 555 Number of Matches: 1

Range 1: 94 to 155

Score	Expect	Identities	Gaps	Strand	Frame
97.1 bits(52)	2e-16()	59/62(95%)	2/62(3%)	Plus/Plus	

```
Query  1    GTGATCC-AATAGGGCCTATGCCTACCCCTGCGATCGCATTTTTAACCGAA-ACATGCGC  58
            ||||||| |||||||||||||||||||||||||||||| |||||||||||| ||||||||
Sbjct  94   GTGATCCAAATAGGGCCTATGCCTACCCCTGCGATCGCGTTTTTAACCGAAGACATGCGC  153

Query  59   TG  60
            ||
Sbjct  154  TG  155
```

Helicobacter pylori strain 595T phosphoglucosamine mutase (glmM) gene, partial cds

Sequence ID: **JN390574.1** Length: 555 Number of Matches: 1

Range 1: 94 to 155

Score	Expect	Identities	Gaps	Strand	Frame
97.1 bits(52)	2e-16()	59/62(95%)	2/62(3%)	Plus/Plus	

```
Query  1    GTGATCC-AATAGGGCCTATGCCTACCCCTGCGATCGCATTTTTAACCGAA-ACATGCGC  58
            ||||||| |||||||||||||||||||||||||||||| |||||||||||| ||||||||
Sbjct  94   GTGATCCAAATAGGGCCTATGCCTACCCCTGCGATCGCGTTTTTAACCGAAGACATGCGC  153

Query  59   TG  60
            ||
Sbjct  154  TG  155
```

Helicobacter pylori strain 590T phosphoglucosamine mutase (glmM) gene, partial cds

Sequence ID: **JN390573.1** Length: 555 Number of Matches: 1

Range 1: 94 to 155

Score	Expect	Identities	Gaps	Strand	Frame
97.1 bits(52)	2e-16()	59/62(95%)	2/62(3%)	Plus/Plus	

```
Query  1    GTGATCC-AATAGGGCCTATGCCTACCCCTGCGATCGCATTTTTAACCGAA-ACATGCGC  58
            ||||||| |||||||||||||||||||||||||||||| |||||||||||| ||||||||
Sbjct  94   GTGATCCAAATAGGGCCTATGCCTACCCCTGCGATCGCTTTTTTAACCGAAGACATGCGC  153

Query  59   TG  60
            ||
Sbjct  154  TG  155
```

Taxonomy

Reports

- ### Lineage

Organism	Blast Name	Score	Number of Hits	Description
Helicobacter	e-proteobacteria		91	
.Helicobacter pylori	e-proteobacteria	97.1	80	**Helicobacter pylori hits**
.Helicobacter pylori SS1	e-proteobacteria	97.1	1	**Helicobacter pylori SS1 hits**
.Helicobacter pylori PMSS1	e-proteobacteria	97.1	1	**Helicobacter pylori PMSS1 hits**
.Helicobacter pylori J99	e-proteobacteria	97.1	2	**Helicobacter pylori J99 hits**
.Helicobacter pylori Gambia94/24	e-proteobacteria	97.1	1	**Helicobacter pylori Gambia94/24 hits**
.Helicobacter pylori NCTC 11637 = CCUG 17874 = ATCC 43504 = JCM 12093	e-proteobacteria	91.6	1	**Helicobacter pylori NCTC 11637 = CCUG 17874 = ATCC 43504 = JCM 12093 hits**
.Helicobacter pylori SJM180	e-proteobacteria	91.6	1	**Helicobacter pylori SJM180 hits**
.Helicobacter pylori B38	e-proteobacteria	91.6	1	**Helicobacter pylori B38 hits**
.Helicobacter pylori 2017	e-proteobacteria	87.9	1	**Helicobacter pylori 2017 hits**
.Helicobacter pylori 2018	e-proteobacteria	87.9	1	**Helicobacter pylori 2018 hits**
.Helicobacter pylori 908	e-proteobacteria	87.9	1	**Helicobacter pylori 908 hits**

- ### Organism

Description	Score	E value	Accession
Helicobacter pylori [e-proteobacteria]			
**Helicobacter pylori strain G-Mx-2003-108 chromosome**	97.1	2e-16	**CP032044**
**Helicobacter pylori strain G-Mx-2006-152 chromosome**	97.1	2e-16	**CP032040**
**Helicobacter pylori DNA, complete genome, strain: PMSS1**	97.1	2e-16	**AP017633**
**Helicobacter pylori strain FDAARGOS_300 chromosome, complete genome**	97.1	2e-16	**CP027404**
**Helicobacter pylori strain oh1 chromosome**	97.1	2e-16	**CP109885**
**Helicobacter pylori strain J99 derivative isolate C2 adapted chromosome, complete genome**	97.1	2e-16	**CP089284**
**Helicobacter pylori strain J99 derivative isolate D1 chromosome, complete genome**	97.1	2e-16	**CP089283**
**Helicobacter pylori strain LIM-009 chromosome, complete genome**	97.1	2e-16	**CP051534**
**Helicobacter pylori strain 603T phosphoglucosamine mutase (glmM) gene, partial cds**	97.1	2e-16	**JN390578**
**Helicobacter pylori strain 600T phosphoglucosamine mutase (glmM) gene, partial cds**	97.1	2e-16	**JN390576**
**Helicobacter pylori strain 595T phosphoglucosamine mutase (glmM) gene, partial cds**	97.1	2e-16	**JN390574**
**Helicobacter pylori strain 599T phosphoglucosamine mutase (glmM) gene, partial cds**	97.1	2e-16	**JN390575**
**Helicobacter pylori strain 590T phosphoglucosamine mutase (glmM) gene, partial cds**	97.1	2e-16	**JN390573**
**Helicobacter pylori strain 370H phosphoglucosamine mutase (glmM) gene, partial cds**	97.1	2e-16	**JN390551**
**Helicobacter pylori strain PP37A1 phosphoglucosamine mutase (glmM) gene, partial cds**	97.1	2e-16	**GU045999**
**Helicobacter pylori strain S23 phosphoglucosamine mutase (glmM) gene, partial cds**	97.1	2e-16	**GU045994**
**Helicobacter pylori strain PS148C1 phosphoglucosamine mutase (glmM) gene, partial cds**	97.1	2e-16	**GU045991**
**Helicobacter pylori strain JF80 phosphoglucosamine mutase (glmM) gene, partial cds**	97.1	2e-16	**AY152929**
**Helicobacter pylori strain PP39A1 phosphoglucosamine mutase (glmM) gene, partial cds**	97.1	2e-16	**AY152913**

Description	Score	E value	Accession
Helicobacter pylori strain PS56C phosphoglucosamine mutase (glmM) gene, partial cds	97.1	2e-16	AY152896
Helicobacter pylori strain S57 phosphoglucosamine mutase (glmM) gene, partial cds	97.1	2e-16	AY152893
Helicobacter pylori strain S55 phosphoglucosamine mutase (glmM) gene, partial cds	97.1	2e-16	AY152892
Helicobacter pylori isolate HE93/10_v1 genome assembly, chromosome: I	93.5	2e-15	LT838273
Helicobacter pylori isolate BCM-300 genome assembly, chromosome: I	93.5	2e-15	LT837687
Helicobacter pylori isolate HE142/09 genome assembly, chromosome: I	93.5	2e-15	LT635478
Helicobacter pylori isolate HE147/09 genome assembly, chromosome: I	93.5	2e-15	LT635477
Helicobacter pylori isolate HE134/09 genome assembly, chromosome: I	93.5	2e-15	LT635476
Helicobacter pylori isolate HE171/09 genome assembly, chromosome: I	93.5	2e-15	LT635474
Helicobacter pylori isolate HE136/09 genome assembly, chromosome: I	93.5	2e-15	LT635473
Helicobacter pylori isolate HE170/09 genome assembly, chromosome: I	93.5	2e-15	LT635472
Helicobacter pylori isolate HE141/09 genome assembly, chromosome: I	93.5	2e-15	LT635471
Helicobacter pylori isolate HE178/09 genome assembly, chromosome: I	93.5	2e-15	LT635460
Helicobacter pylori isolate HE132/09 genome assembly, chromosome: I	93.5	2e-15	LT635459
Helicobacter pylori isolate HE143/09 genome assembly, chromosome: I	93.5	2e-15	LT635458
Helicobacter pylori isolate HE101/09 genome assembly, chromosome: I	93.5	2e-15	LT635456
Helicobacter pylori strain G-Mx-2005-108 chromosome	91.6	8e-15	CP032042
Helicobacter pylori strain G-Mx-2011-124 chromosome	91.6	8e-15	CP032038
Helicobacter pylori strain C-Mx-2011-171 chromosome	91.6	8e-15	CP032033
Helicobacter pylori strain C-Mx-2010-8 chromosome	91.6	8e-15	CP032027
Helicobacter pylori strain B147 chromosome, complete genome	91.6	8e-15	CP024946
Helicobacter pylori strain B140 chromosome, complete genome	91.6	8e-15	CP024948
Helicobacter pylori strain 24-A-EK1 chromosome, complete genome	91.6	8e-15	CP032907
Helicobacter pylori strain 478-A-EK1 chromosome, complete genome	91.6	8e-15	CP032899
Helicobacter pylori strain HP14039 chromosome, complete genome	91.6	8e-15	CP034147
Helicobacter pylori DNA, complete genome, strain: ATCC43504	91.6	8e-15	AP017632
Helicobacter pylori strain FDAARGOS_298 chromosome, complete genome	91.6	8e-15	CP028325
Helicobacter pylori strain 444A6 chromosome, complete genome	91.6	8e-15	CP117024
Helicobacter pylori strain K262 chromosome, complete genome	91.6	8e-15	CP091772
Helicobacter pylori strain MT5118 chromosome, complete genome	91.6	8e-15	CP071975
Helicobacter pylori strain MT5125 chromosome, complete genome	91.6	8e-15	CP071978
Helicobacter pylori strain MT5111 chromosome, complete genome	91.6	8e-15	CP071981
Helicobacter pylori strain MT5135 chromosome, complete genome	91.6	8e-15	CP071982
Helicobacter pylori strain MT5114 chromosome, complete genome	91.6	8e-15	CP071984
Helicobacter pylori strain MT5119 chromosome, complete genome	91.6	8e-15	CP071985
Helicobacter pylori strain LIM-001 chromosome, complete genome	91.6	8e-15	CP051541
Helicobacter pylori strain LIM-010 chromosome, complete genome	91.6	8e-15	CP051533
Helicobacter pylori strain 584T phosphoglucosamine mutase (glmM) gene, partial cds	91.6	8e-15	JN390572
Helicobacter pylori strain 2161N phosphoglucosamine mutase (glmM) gene, partial cds	91.6	8e-15	JN390567
Helicobacter pylori strain 2133N phosphoglucosamine mutase (glmM) gene, partial cds	91.6	8e-15	JN390566
Helicobacter pylori HpKE21 DNA, complete genome	91.6	8e-15	AP023320
Helicobacter pylori strain PP2B4 phosphoglucosamine mutase (glmM) gene, partial cds	91.6	8e-15	GU046000
Helicobacter pylori strain PP23A1 phosphoglucosamine mutase (glmM) gene, partial cds	91.6	8e-15	AY152914
Helicobacter pylori strain PP22B2 phosphoglucosamine mutase (glmM) gene, partial cds	91.6	8e-15	AY152911
Helicobacter pylori strain PS184A phosphoglucosamine mutase (glmM) gene, partial cds	91.6	8e-15	AY152903
Helicobacter pylori strain S49 phosphoglucosamine mutase (glmM) gene, partial cds	91.6	8e-15	AY152890
Helicobacter pylori strain GCT 43 chromosome, complete genome	87.9	1e-13	CP048600
Helicobacter pylori strain C-Mx-2010-97 chromosome	87.9	1e-13	CP032036
Helicobacter pylori strain C-Mx-2010-3 chromosome	87.9	1e-13	CP032023

Description	Score	E value	Accession
**Helicobacter pylori strain C-Mx-2010-5 chromosome**	87.9	1e-13	**CP032022**
**Helicobacter pylori strain MT5105 chromosome, complete genome**	87.9	1e-13	**CP071977**
**Helicobacter pylori strain MT5124 chromosome, complete genome**	87.9	1e-13	**CP071986**
**Helicobacter pylori strain MT5136 chromosome, complete genome**	87.9	1e-13	**CP071987**
**Helicobacter pylori strain PUNO-003 chromosome, complete genome**	87.9	1e-13	**CP051500**
**Helicobacter pylori strain PUNO-009 chromosome, complete genome**	87.9	1e-13	**CP051495**
**Helicobacter pylori strain PS184A1 phosphoglucosamine mutase (glmM) gene, partial cds**	87.9	1e-13	**GU045992**
**Helicobacter pylori strain PP25A1 phosphoglucosamine mutase (glmM) gene, partial cds**	87.9	1e-13	**AY152915**
**Helicobacter pylori strain PP01A1 phosphoglucosamine mutase (glmM) gene, partial cds**	87.9	1e-13	**AY152909**
**Helicobacter pylori strain PS158A1 phosphoglucosamine mutase (glmM) gene, partial cds**	87.9	1e-13	**AY152900**
**Helicobacter pylori strain S22 phosphoglucosamine mutase (glmM) gene, partial cds**	87.9	1e-13	**AY152885**
**H.pylori urease (ureA, ureB, ureC, ureD) genes, complete cds**	87.9	1e-13	**M60398**
Helicobacter pylori SS1 [e-proteobacteria]			
**Helicobacter pylori SS1, complete genome**	97.1	2e-16	**CP009259**
Helicobacter pylori PMSS1 [e-proteobacteria]			
**Helicobacter pylori strain PMSS1 complete genome**	97.1	2e-16	**CP018823**
Helicobacter pylori J99 [e-proteobacteria]			
**Helicobacter pylori J99, complete genome**	97.1	2e-16	**CP011330**
**Helicobacter pylori J99, complete genome**	97.1	2e-16	**AE001439**
Helicobacter pylori Gambia94/24 [e-proteobacteria]			
**Helicobacter pylori Gambia94/24, complete genome**	97.1	2e-16	**CP002332**
Helicobacter pylori NCTC 11637 = CCUG 17874 = ATCC 43504 = JCM 12093 [e-proteobacteria]			
**Helicobacter pylori strain NCTC11637 genome assembly, chromosome: 1**	91.6	8e-15	**LS483488**
Helicobacter pylori SJM180 [e-proteobacteria]			
**Helicobacter pylori SJM180, complete genome**	91.6	8e-15	**CP002073**
Helicobacter pylori B38 [e-proteobacteria]			
**Helicobacter pylori B38 complete genome, strain B38**	91.6	8e-15	**FM991728**
Helicobacter pylori 2017 [e-proteobacteria]			
**Helicobacter pylori 2017, complete genome**	87.9	1e-13	**CP002571**
Helicobacter pylori 2018 [e-proteobacteria]			
**Helicobacter pylori 2018, complete genome**	87.9	1e-13	**CP002572**
Helicobacter pylori 908 [e-proteobacteria]			
**Helicobacter pylori 908, complete genome**	87.9	1e-13	**CP002184**

- ## Taxonomy

Taxonomy	Number of hits	Number of Organisms	Description
Helicobacter	91	11	
. Helicobacter pylori	80	11	**Helicobacter pylori hits**
.. Helicobacter pylori SS1	1	1	**Helicobacter pylori SS1 hits**
.. Helicobacter pylori PMSS1	1	1	**Helicobacter pylori PMSS1 hits**
.. Helicobacter pylori J99	2	1	**Helicobacter pylori J99 hits**
.. Helicobacter pylori Gambia94/24	1	1	**Helicobacter pylori Gambia94/24 hits**
.. Helicobacter pylori NCTC 11637 = CCUG 17874 = ATCC 43504 = JCM 12093	1	1	**Helicobacter pylori NCTC 11637 = CCUG 17874 = ATCC 43504 = JCM 12093 hits**
.. Helicobacter pylori SJM180	1	1	**Helicobacter pylori SJM180 hits**
.. Helicobacter pylori B38	1	1	**Helicobacter pylori B38 hits**
.. Helicobacter pylori 908	1	3	**Helicobacter pylori 908 hits**
... Helicobacter pylori 2017	1	1	**Helicobacter pylori 2017 hits**
... Helicobacter pylori 2018	1	1	**Helicobacter pylori 2018 hits**
